# Evaluation of divergent yeast genera for fermentation-associated stresses and identification of a robust sugarcane distillery waste isolate *Saccharomyces cerevisiae* NGY10 for lignocellulosic ethanol production in SHF and SSF

**DOI:** 10.1186/s13068-019-1379-x

**Published:** 2019-02-27

**Authors:** Ajay Kumar Pandey, Mohit Kumar, Sonam Kumari, Priya Kumari, Farnaz Yusuf, Shaik Jakeer, Sumera Naz, Piyush Chandna, Ishita Bhatnagar, Naseem A. Gaur

**Affiliations:** Yeast Biofuel Group, DBT-ICGEB Center for Advanced Bioenergy Research, International Center for Genetic Engineering and Biotechnology (ICGEB), New Delhi, 110067 India

**Keywords:** Thermo-tolerance, Inhibitors, SHF, SSF, Fermentation, Ethanol

## Abstract

**Background:**

Lignocellulosic hydrolysates contain a mixture of hexose (C6)/pentose (C5) sugars and pretreatment-generated inhibitors (furans, weak acids and phenolics). Therefore, robust yeast isolates with characteristics of C6/C5 fermentation and tolerance to pretreatment-derived inhibitors are pre-requisite for efficient lignocellulosic material based biorefineries. Moreover, use of thermotolerant yeast isolates will further reduce cooling cost, contamination during fermentation, and required for developing simultaneous saccharification and fermentation (SSF), simultaneous saccharification and co-fermentation (SScF), and consolidated bio-processing (CBP) strategies.

**Results:**

In this study, we evaluated thirty-five yeast isolates (belonging to six genera including *Saccharomyces, Kluyveromyces, Candida*, *Scheffersomyces, Ogatea* and *Wickerhamomyces*) for pretreatment-generated inhibitors {furfural, 5-hydroxymethyl furfural (5-HMF) and acetic acid} and thermotolerant phenotypes along with the fermentation performances at 40 °C. Among them, a sugarcane distillery waste isolate, *Saccharomyces cerevisiae* NGY10 produced maximum 49.77 ± 0.34 g/l and 46.81 ± 21.98 g/l ethanol with the efficiency of 97.39% and 93.54% at 30 °C and 40 °C, respectively, in 24 h using glucose as a carbon source. Furthermore, isolate NGY10 produced 12.25 ± 0.09 g/l and 7.18 ± 0.14 g/l of ethanol with 92.81% and 91.58% efficiency via SHF, and 30.22 g/l and 25.77 g/l ethanol with 86.43% and 73.29% efficiency via SSF using acid- and alkali-pretreated rice straw as carbon sources, respectively, at 40 °C. In addition, isolate NGY10 also produced 92.31 ± 3.39 g/l (11.7% v/v) and 33.66 ± 1.04 g/l (4.26% v/v) ethanol at 40 °C with the yields of 81.49% and 73.87% in the presence of 30% w/v glucose or 4× concentrated acid-pretreated rice straw hydrolysate, respectively. Moreover, isolate NGY10 displayed furfural- (1.5 g/l), 5-HMF (3.0 g/l), acetic acid- (0.2% v/v) and ethanol-(10.0% v/v) tolerant phenotypes.

**Conclusion:**

A sugarcane distillery waste isolate NGY10 demonstrated high potential for ethanol production, C5 metabolic engineering and developing strategies for SSF, SScF and CBP.

**Electronic supplementary material:**

The online version of this article (10.1186/s13068-019-1379-x) contains supplementary material, which is available to authorized users.

## Background

Fossil fuels are the mainstay of global transport fuel; nonetheless, their incessant depletion and climate deterioration conduct led to a rigorous search for low carbon emitting alternative fuel sources [1, 2]. Liquid transport fuel, bioethanol, does not add extra carbon to the environment and its compatibility with the existing internal combustion (IC) engines makes it preferred green fuel worldwide [3, 4]. For cost-effective lignocellulosic ethanol production, low-cost feedstock is one of the most important components. India generates about 650 million metric tons of lignocellulosic waste annually through routine agricultural activities, favored their optimum exploitation as a low-cost renewable carbon source for biofuel production [5–7].

Lignocellulosic biomass to ethanol conversion requires three separate processes such as pretreatment, saccharification and fermentation [8, 9]. These separate processes increase the cost of lignocellulosic ethanol production as compared to the first-generation biofuel, wherein pretreatment and saccharification are not required. Therefore, developing technologies to combine the separate process is of great interest. Simultaneous Saccharification and Fermentation (SSF) is an attractive strategy involving single reactor, lowering the capital cost by minimizing the quantity of reactor vessel, energy input, contamination risk, product inhibition and processing time [1, 10–12]. Nonetheless, the major constraint of SSF is the misalliance of thermal optima of enzymatic saccharification (~ 45–50 °C) and fermentation (~ 30 °C) [1]; therefore, an intermediate temperature of ~ 40 °C for SSF is suggested to meet the thermal alliance of saccharification and fermentation process [11, 13–15].

*Saccharomyces cerevisiae* is a preferred workhorse for corn/sugarcane ethanol industry [14, 16]. Nonetheless, the fermentation of lignocellulosic hydrolysate (LH) is challenging, because it contains C5 sugars along with the C6, which is not a preferential sugar for *S. cerevisiae* [4, 11]. In addition, LH also contains pretreatment-generated toxic byproducts such as furfural, 5-hydroxymethyl furfural (5-HMF), acetic acid and phenolics, which reduces the growth and fermentation performances of microorganisms [2, 17, 18]. Although, some studies have suggested detoxification (inhibitors removal) of LH through overliming, treatment with activated charcoal, hydrophobic/anion exchange resin and laccase, but these increase the overall production cost due to the requirement of the additional process and lead to sugar loss [19].

Although, in recent years, many yeast strains with improved lignocellulosic ethanol production performances and pretreatment-generated inhibitor-tolerant phenotypes have been isolated [6, 20, 21] or developed [22–24], but efficient C6/C5 fermentation at 40 °C in the presence of pretreatment generated inhibitors is still a challenging task and need to be addressed. Therefore, search for novel yeast isolates with desired characteristics of industrial lignocellulosic ethanol production is a continuous process over decades. In our previous study, we evaluated the fermentation and inhibitor tolerance performances of yeast isolates procured from the Microbial Type culture collection (MTTC), Chandigarh, India [25]. In this study, we explored the natural habitats such as distillery waste, dairy waste, hot springs, sewage and algal bloom for identification of robust yeast isolates.

In most of the previous studies, yeast species belonging to one or two genera were evaluated for thermotolerance, pre-treatment inhibitor tolerance and LH fermentations, simultaneously [4, 6, 26–28]. In this study, yeast isolates belonging to *Saccharomyces*, *Kluyveromyces, Candida*, *Scheffersomyces, Ogatea* and *Wickerhamomyces* genera were evaluated for fermentation performances at 40 °C along with the pretreatment generated inhibitors (furfural, 5-HMF and acetic acid) and fermentation stress-tolerant phenotypes. We also evaluated the sugar assimilation profile and fermentation performances of selected isolates at 30 °C and 40 °C using different carbon sources (glucose, xylose, and rice straw hydrolysates) via SHF and SSF processes.

## Results

### Isolation and molecular characterization of yeast isolates

**Fig. 1:**
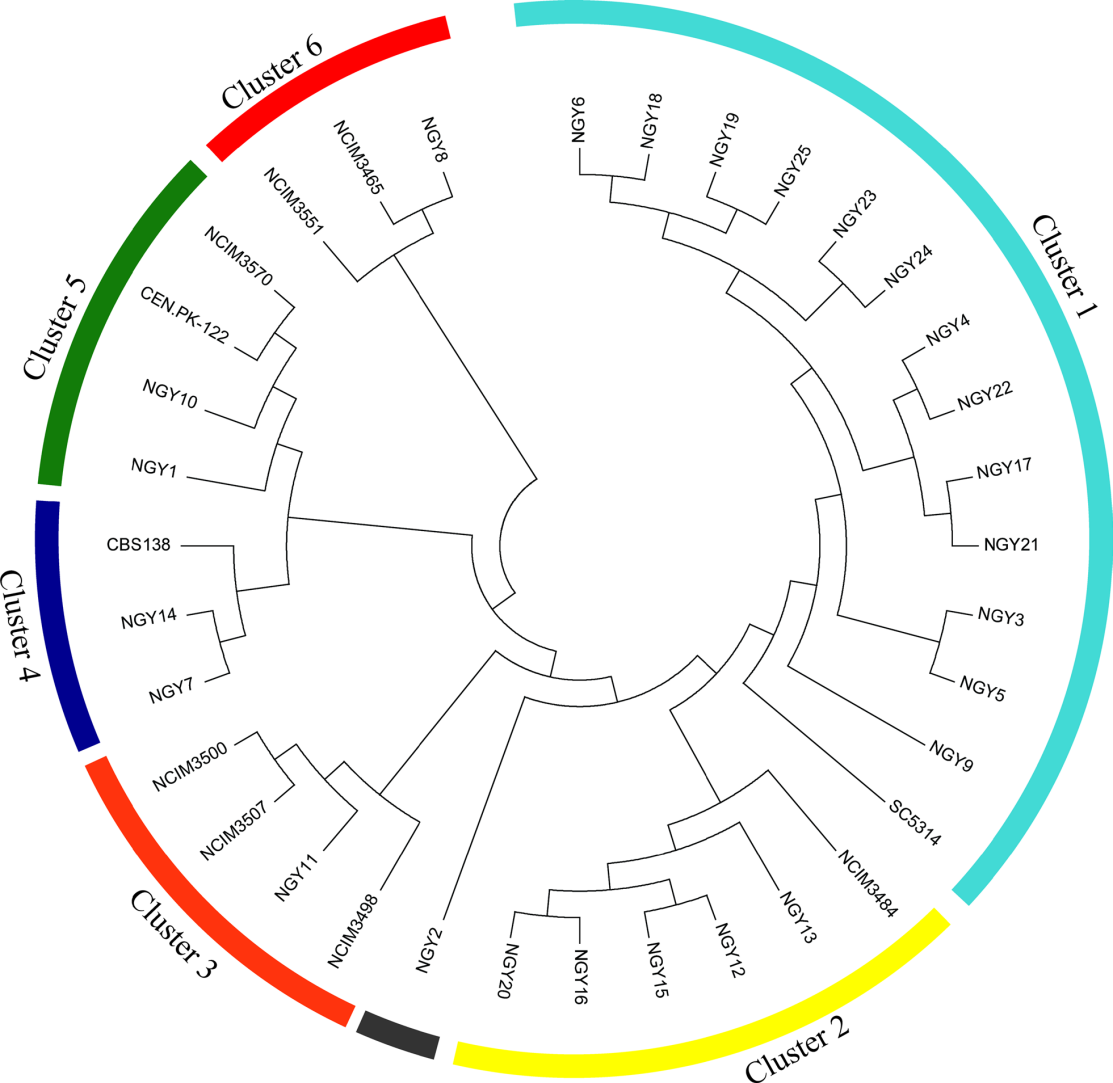
Phylogenetic tree displaying evolutionary relationship among yeast isolates based on ITS sequences. ITS sequences were aligned by ClustalW (a multiple sequence alignment tool) and phylogenetic analysis was performed by MEGA 6.0 software using maximum likelihood method with bootstrap value 1000 and Tamura-Nei model. Cluster 1: *C. tropicalis* isolates (NGY21, NGY22, NGY19, NGY18, NGY17, NGY9, NGY6, NGY5, NGY4, NGY3, NGY23, NGY24 and NGY25) and *C. albicans* isolate SC5314; Cluster 2: *C. lusitaniae* isolate NCIM3484 and *P. kudriavzevii* isolates (NGY12, NGY13, NGY15, NGY16 and NGY20); Cluster 3: *C. sehatae* isolate NCIM3500, *S. stipitis* isolates (NCIM3507 and NCIM3498) and *O. thermophilla* isolate NGY11; Cluster 4: *C. glabrata* isolates (NGY7, NGY14 and CBS138); Cluster 5: *S. cerevisiae* isolates (CEN.PK-122, NGY1, NGY10 and NCIM3570) and Cluster 6: *Kluyveromyces* sp. isolates (NGY8, NCIM3465 and NCIM3551). *W. anomalus* isolate NGY2 did not cluster with any other yeast sp

More than 500 microbial colonies showing yeast-like growth were isolated from serial dilutions (10^−1^–10^−6^) of six different samples ("Methods"). 82 yeast looking colonies of 10^−3^ and 10^−4^ dilutions were further screened on chrome agar [29]. Based on the chrome agar screening and growth at 40 °C, 25 yeast-like colonies were selected to evaluate their fermentation potential related to lignocellulosic ethanol production. These yeast-looking colonies were identified by Internal transcribed spacer (ITS) sequencing followed by National Center for Biotechnology Information (NCBI) nucleotide Basic Local Alignment Search Tool (BLAST) analysis. Based on the NCBI database similarity index, these colonies belonged to six genera including *Saccharomyces*, *Kluyveromyces, Candida*, *Scheffersomyces, Ogatea* and *Wickerhamomyces* (Table 1). To further enhance the yeast genetic diversity in this study, we included nine uncharacterized C6 and C5 utilizing yeast isolates belonging to different genera (procured from the National culture collection of industrial microorganisms (NCIM), Pune, India) along with the two industrial strains *S. cerevisiae* CEN.PK-122 and Angel yeast (Table 1). CEN.PK-122 and Angel Yeast have been shown as industrial reference strains in several previous studies [30–36]. Next, we studied the Phylogenetic relationship among these isolates based on ITS sequences. As shown by the phylogenetic tree (Fig. 1), all isolates grouped into six clusters. As expected, in agreement with the previous study [37], *C. tropicalis* isolates (NGY21, NGY22, NGY19, NGY18, NGY17, NGY9, NGY6, NGY5, NGY4, NGY3, NGY23, NGY24 and NGY25) displayed phylogenetic closeness to *C. albicans* strain SC5314 in cluster 1. Whereas, other *Candida* species, *C. glabrata* (cluster 4: including isolates NGY7, NGY14 and CBS138) were closer to the *S. cerevisiae* (cluster 5: including isolates CEN.PK-122, NGY1, NGY10 and NCIM3570) and *Kluyveromyces* sp. (cluster 6: including isolates NGY8, NCIM3465 and NCIM3551). Interestingly, other *Candida* species, *C. lusitaniae* isolate NCIM3484 shared cluster 2, with five isolates of *P. kudriavzevii* (NGY12, NGY13, NGY15, NGY16 and NGY20), while another species *C. sehatae* isolate NCIM3500 displayed closeness to *S. stipitis* isolates (NCIM3507 and NCIM3498) and *O. thermophilla* isolate NGY11 in cluster 3. These results suggesting the existence of most diverge characteristics in *Candida* among all tested genera. An isolate NGY2, belonging to *W. anomalus* species, did not cluster with other isolates in this study. Together, as expected, isolates belonging to the same genus were closer as compared to the isolates of different genera. Notably, *C. glabrata, S. cerevisiae*, and *Kluyveromyces* sp. isolates were grouped into cluster 4, cluster 5 and cluster 6, respectively, but originated from same nodal point. Hence, they are phylogenetically close to each other as compared to other yeasts in this study. Since, *S. cerevisiae* is known for bioethanol production, it is expected that phylogenetically closer yeast isolates such as *C. glabrata* and *Kluyveromyces* sp. may also possess high potential for bioethanol production.

**Table 1:** List of selected yeast isolates used in this study and their source

S. no.	Yeast strains	Source
1.	Angel yeast	Angel Active Dry Ethanol Yeast, Angel Yeast Co. Ltd., Hubei, China
2.	*S. cerevisiae* CEN-PK-122	Gifted from Peter Kotter, J. W. Goethe Universitat Frankfurt, Germany
3.	*S. cerevisiae* NCIM 3570	Procured from National Collection of Industrial Microorganisms (NCIM), Pune, India
4.	*S. cerevisiae* NGY1	Isolated from sugarcane distillery waste, Bijnor, Uttar Pradesh, India
5.	*S. cerevisiae* NGY10	Isolated from sugarcane distillery waste, Bulandshahr, Uttar Pradesh, India
6.	*K. marxianus* NCIM 3465	Procured from National Collection of Industrial Microorganisms (NCIM), Pune, India
7.	*K. marxianus* NGY8	Isolated from Mother Dairy waste, New Delhi, India
8.	*K. lactis* NCIM 3551	Procured from National Collection of Industrial Microorganisms (NCIM), Pune, India
9.	*S. stipitis* NCIM 3507	Procured from National Collection of Industrial Microorganisms (NCIM), Pune, India
10.	*S. stipitis* NCIM 3498	Procured from National Collection of Industrial Microorganisms (NCIM), Pune, India
11.	*C. shehatae* NCIM 3500	Procured from National Collection of Industrial Microorganisms (NCIM), Pune, India
12.	*C. lusitaniae* NCIM 3484	Procured from National Collection of Industrial Microorganisms (NCIM), Pune, India
13.	*C. albicans* SC5314	Gifted from Prof. Rajendra Prasad, Laboratory of Membrane Biology, Jawahar Lal Nehru University, New Delhi
14.	*W. anomalus* NGY2	Isolated from sugarcane distillery waste, Bijnor, Uttar Pradesh, India
15.	*O. thermophila* NGY11	Isolated from sugarcane distillery waste, Bulandshahr, Uttar Pradesh, India
16.	*C. glabrata* CBS138	Gifted from Prof. Neeraj Chauhan, Department of Microbiology, Biochemistry and Molecular Genetics, Rutgers New Jersey Medical School
17.	*C. glabrata* NGY7	Isolated from sugarcane distillery waste, Bijnor, Uttar Pradesh, India
18.	*C. glabrata* NGY14	Isolated from sewage and algal bloom, Bulandshahr, Uttar Pradesh, India
19.	*P. kudriavzevii* NGY12	Isolated from sugarcane distillery waste, Bulandshahr, Uttar Pradesh, India
20.	*P. kudriavzevii* NGY13	Isolated from sugarcane distillery waste, Bulandshahr, Uttar Pradesh, India
21.	*P. kudriavzevii* NGY15	Isolated from sewage and algal bloom, Bulandshahr, Uttar Pradesh, India
22.	*P. kudriavzevii* NGY16	Isolated from sewage and algal bloom, Bulandshahr, Uttar Pradesh, India
23.	*P. kudriavzevii* NGY20	Isolated from sugarcane distillery waste, Bhopal, Madhya Pradesh, India
24.	*C. dubliniensis* NGY5	Isolated from sugarcane distillery waste, Bijnor, Uttar Pradesh, India
25.	*C. tropicalis* NGY3	Isolated from sugarcane distillery waste, Bijnor, Uttar Pradesh, India
26.	*C. tropicalis* NGY4	Isolated from sugarcane distillery waste, Bijnor, Uttar Pradesh, India
27.	*C. tropicalis* NGY6	Isolated from sugar distillery waste, Bijnor, Uttar Pradesh, India
28.	*C. tropicalis* NGY9	Isolated from sugarcane distillery waste, Dhampur, Uttar Pradesh, India
29.	*C. tropicalis* NGY17	Isolated from sewage and algal bloom, Bulandshahr, Uttar Pradesh, India
30.	*C. tropicalis* NGY18	Isolated from sewage and algal bloom, Bulandshahr, Uttar Pradesh, India
31.	*C. tropicalis* NGY19	Isolated from sewage and algal bloom, Bulandshahr, Uttar Pradesh, India
32.	*C. tropicalis* NGY21	Isolated from sugarcane distillery waste, Bhopal Madhya Pradesh, India
33.	*C. tropicalis* NGY22	Isolated from sugarcane distillery waste, Bhopal Madhya Pradesh, India
34.	*C. tropicalis* NGY23	Isolated from sugarcane distillery waste, Bhopal Madhya Pradesh, India
35.	*C. tropicalis* NGY24	Isolated from sugarcane distillery waste, Bhopal Madhya Pradesh, India
36.	*C. tropicalis* NGY25	Isolated from sugarcane distillery waste, Bhopal Madhya Pradesh, India

*S. cerevisiae, Saccharomyces cerevisiae*; *K. marxianus*, *Kluyveromyces marxianus*; *K. lactis*, *Kluyveromyces lactis*; *S. stipitis*, *Scheffersomyces stipitis*; *C. shehatae, Candida shehatae*; *C. lusitaniae*, *Candida lusitaniae*; *C. albicans*, *Candida albicans*; *W. anomalus, Wickerhamomyces anomalus*; *O. thermophila, Ogatea thermophila*; *C. glabrata, Candida glabrata*; *P. kudriavzevii, Pichia kudriavzevii*; *C. dubliniensis, Candida dubliniensis* and *C. tropicalis*, *Candida tropicalis*

### Thermotolerance profile

Thermotolerant phenotypes of the yeast isolates were determined by comparative kinetic growth analysis such as doubling time (*T*_d_) and specific growth rate (*μ*) at 30 °C and 40 °C, respectively. Isolates displaying less than 5.0% increase in *T*_d_ at 40 °C as compared to 30 °C were considered as thermotolerant (Table 2). Among all the tested isolates NGY10, NCIM3465, NGY8, NGY7, NGY15, NGY20, NGY3, NGY5, NGY21 and NGY25 displayed thermotolerant phenotypes. Wherein, isolates NGY8, NCIM 3465, NGY20, NGY7 and NGY10 were fastest growing with less than 5% increase in *T*_d_ at 40 °C with 56.65 ± 0.98 min, 59.10 ± 0.93 min, 59.48 ± 1.72 min, 68.05 ± 3.47 min and 76.014 ± 1.13 min, respectively, and all other isolates displayed between 5 and 20% increase in *T*_d_. Among them, isolates NCIM3498, NCIM3507 and NCIM3500 displayed the slowest growth with maximum increase in *T*_d_ of 14.39%, 19.74% and 15.08%, respectively, at 40 °C as compared to 30 °C (Table 2).

**Table 2:** Specific growth rate and doubling time of yeast isolates at 30 °C and 40 °C in YEPD medium

S. no.	Yeast strains	30 °C		40 °C		% increase in *T*_d_ at 40 °C	% decrease in µ at 40 °C
		Doubling time (*T*_d_), (min)	Specific growth rate (µ) (h^−1^)	Doubling time (*T*_d_) (min)	Specific growth rate (µ) (h^−1^)		
1.	Angel yeast	75.21 ± 1.63	0.552 ± 0.05	81.63 ± 1.06	0.509 ± 0.05	8.53	7.78
2.	*S. cerevisiae* CEN-PK-122	91.18 ± 1.06^a^	0.456 ± 0.02	100.05 ± 1.7	0.415 ± 0.05	9.72	8.86
3.	*S. cerevisiae* NCIM 3570	98.07 ± 1.59	0.424 ± 0.03	108.6 ± 1.5	0.383 ± 0.06	10.7	9.67
4.	*S. cerevisiae* NGY1	85.56 ± 0.53	0.486 ± 0.01	90.98 ± 0.49	0.457 ± 0.04	6.34	5.97
5.	*S. cerevisiae* NGY10	73.20 ± 1.21	0.568 ± 0.023	76.014 ± 1.13	0.547 ± 0.05	3.84	3.69
6.	*K. marxianus* NCIM 3465	57.04 ± 1.01	0.729 ± 0.019	59.10 ± 0.93	0.704 ± 0.04	3.55	3.43
7.	*K. marxianus* NGY8	55.0 ± 1.06	0.756 ± 0.02	56.65 ± 0.98	0.734 ± 0.04	2.99	2.91
8.	*K. lactis* NCIM 3551	92.4 ± 1.64	0.45 ± 0.031	97.83 ± 1.51	0.425 ± 0.05	5.88	5.56
9.	*S. stipitis* NCIM 3507	89.04 ± 0.42	0.467 ± 0.008	106.61 ± 0.39	0.39 ± 0.029	19.74	16.49
10.	*S. stipitis* NCIM 3498	95.15 ± 0.37	0.437 ± 0.007	108.84 ± 0.34	0.382 ± 0.03	14.39	12.58
11.	*C. shehatae* NCIM 3500	83.83 ± 2.65	0.496 ± 0.05	96.50 ± 2.45	0.431 ± 0.07	15.08	13.10
12.	*C. lusitaniae* NCIM 3484	89.04 ± 0.95	0.467 ± 0.018	97.61 ± 0.88	0.426 ± 0.04	9.62	8.77
13.	*C. albicans* SC5314	76.01 ± 1.6	0.547 ± 0.031	82.01 ± 1.52	0.507 ± 0.05	7.9	7.31
14.	*W. anomalus* NGY2	78.01 ± 1.53	0.533 ± 0.029	84.0 ± 1.42	0.495 ± 0.02	7.68	7.13
15.	*O. thermophila* NGY11	93.86 ± 1.37	0.443 ± 0.026	98.80 ± 1.3	0.421 ± 0.01	5.23	4.97
16.	*C. glabrata* CBS138	62.06 ± 1.06	0.67 ± 0.02	67.20 ± 0.98	0.619 ± 0.01	8.24	7.61
17.	*C. glabrata* NGY7	65.07 ± 3.76	0.639 ± 0.071	68.05 ± 3.47	0.611 ± 0.06	4.58	4.38
18.	*C. glabrata* NGY14	68.05 ± 2.12	0.611 ± 0.04	72.69 ± 1.96	0.572 ± 0.03	6.81	6.38
19.	*P. kudriavzevii* NGY12	62.34 ± 2.58	0.667 ± 0.06	68.05 ± 2.94	0.611 ± 0.05	9.16	8.39
20.	*P. kudriavzevii* NGY13	59.74 ± 1.65	0.696 ± 0.091	62.06 ± 3.00	0.67 ± 0.06	6.86	6.42
21.	*P. kudriavzevii* NGY15	61.42 ± 1.08	0.677 ± 0.02	65.07 ± 0.98	0.639 ± 0.043	4.85	4.63
22.	*P. kudriavzevii* NGY16	60.88 ± 2.42	0.683 ± 0.045	66.85 ± 2.21	0.622 ± 0.012	7.72	7.16
23.	*P. kudriavzevii* NGY20	57.83 ± 2.38	0.719 ± 0.035	59.48 ± 1.72	0.699 ± 0.07	4.85	4.62
24.	*C. dubliniensis* NGY5	81.05 ± 0.63	0.513 ± 0.012	84.0 ± 0.6	0.495 ± 0.045	3.63	3.51
25.	*C. tropicalis* NGY3	80.12 ± 1.16	0.519 ± 0.022	83.16 ± 1.1	0.5 ± 0.055	3.8	3.66
26.	*C. tropicalis* NGY4	84.03 ± 3.28	0.495 ± 0.062	88.47 ± 3.04	0.47 ± 0.095	5.32	5.05
27.	*C. tropicalis* NGY6	82.33 ± 1.48	0.505 ± 0.028	87.17 ± 1.37	0.477 ± 0.061	5.87	5.54
28.	*C. tropicalis* NGY9	79.05 ± 1.0	0.526 ± 0.019	84.0 ± 0.93	0.495 ± 0.052	6.26	5.89
29.	*C. tropicalis* NGY17	80.12 ± 4.3	0.519 ± 0.081	85.03 ± 3.96	0.489 ± 0.052	6.13	5.78
30.	*C. tropicalis* NGY18	75.05 ± 2.96	0.554 ± 0.056	81.10 ± 2.74	0.513 ± 0.052	7.99	7.4
31.	*C. tropicalis* NGY19	78.01 ± 3.34	0.533 ± 0.063	84.0 ± 3.08	0.495 ± 0.052	7.67	7.13
32.	*C. tropicalis* NGY21	84.0 ± 3.13	0.495 ± 0.059	86.81 ± 2.89	0.479 ± 0.052	3.34	3.23
33.	*C. tropicalis* NGY22	80.11 ± 1.54	0.519 ± 0.029	85.03 ± 1.42	0.489 ± 0.052	6.13	5.78
34.	*C. tropicalis* NGY23	84.0 ± 4.24	0.495 ± 0.08	90.98 ± 3.92	0.457 ± 0.121	8.32	7.67
35.	*C. tropicalis* NGY24	81.05 ± 4.87	0.513 ± 0.092	88.09 ± 4.51	0.472 ± 0.133	8.68	7.99
36.	*C. tropicalis* NGY25	82.33 ± 1.32	0.505 ± 0.025	86.09 ± 1.22	0.483 ± 0.066	4.55	4.36

^a^Mean ± standard deviation, Experiments were conducted in biological triplicates (*n* = 3) and value presented in table are their mean values with standard deviation

### Sugar assimilation profile

Glucose and xylose are the most abundant sugars in the lignocellulosic hydrolysates (LH), while other mono-saccharides (galactose, mannose and arabinose) and di-saccharides (cellobiose) are present in trace amount [38, 39]. Therefore, fermentation of all the sugars present in the LH is important to develop an economically viable lignocellulosic ethanol production technology. Hence, we analyzed the carbon source utilization potential of yeast isolates with pentoses, hexoses and disaccharides, individually (Table 3). All the tested isolates displayed growth on glucose and mannose and all isolates except NGY8, NCIM3500, SC5314, CBS138 and NGY14 also displayed growth on galactose. As expected, none of the *S. cerevisiae* isolates displayed growth on xylose (Table 3), whereas isolates NGY8, NCIM3465, NCIM 3498, NCIM3507, NCIM3500, NCIM3484, NGY2, NGY11, NGY7, NGY12, NGY13, NGY15, NGY16, NGY20, NGY5 and *C. tropicalis* isolates were able to grow on xylose. The growth on xylose suggested functionally active xylose metabolic pathway and transporter in these isolates. Interestingly, two isolates NGY8 and NGY11 were also able to utilize arabinose as a carbon source, suggesting the presence of the arabinose transporter and functionally active arabinose metabolic pathway in these isolates. Moreover, isolates NGY8, NCIM3507, NCIM3484, NGY2, NGY11, NGY3 and NGY19 were able to utilize cellobiose as a carbon source. Cellobiose is broken down into glucose by functionally active β-glucosidase. Isolates NCIM3465, NGY8, NCIM3551, NCIM3500 and NGY4 also displayed the ability to utilize lactose, indicating the expression of lactose catabolizing genes in these isolates. However, all the isolates displayed growth on maltose except NGY8, SC5314 and NGY14.

**Table 3:** Sugar assimilation profile of yeast isolates at 30 °C in SD medium

S. no.	Yeast strains	C6 sugars			C5 sugars		Disaccharides		
		Glucose	Mannose	Galactose	Xylose	Arabinose	Cellobiose	Lactose	Maltose
1.	Angel yeast	++	+ N	+ N	−	−	−	−	+ N
2.	*S. cerevisiae* CEN-PK-122	++ ^a^	+ N^b^	+ N	−	−	−	−	+ N
3.	*S. cerevisiae* NCIM 3570	++	+ N	+ N	−	−	−	−	+ N
4.	*S. cerevisiae* NGY1	++	+ N	+ N	−	−	−	−	+ N
5.	*S. cerevisiae* NGY10	++	+ N	+ N	−	−	−	−	+ N
6.	*K. marxianus* NCIM 3465	++	+ N	−^c^	++	−	−	+ N	+ N
7.	*K. marxianus* NGY8	++	+ N	+ N	++	+N	+ N	+ N	−
8.	*K. lactis* NCIM 3551	++	+ N	+ N	−	−	−	+ N	+ N
9.	*S. stipitis* NCIM 3507	+ +	+ N	+ N	++	−	+ N	−	+ N
10.	*S. stipitis* NCIM 3498	+ +	+ N	+ N	++	−	−	−	+ N
11.	*C. shehatae* NCIM 3500	++	+ N	−	++	−	−	+ N	+ N
12.	*C. lusitaniae* NCIM 3484	++	+ N	+ N	++	−	+ N	−	+ N
13.	*C. albicans* SC5314	++	+ N	−	+ −^d^	−	−	−	−
14.	*W. anomalus* NGY2	++	+ N	+ N	++	−	+ N	−	+ N
15.	*O. thermophila* NGY11	++	+ N	+ N	++	+ N	+ N	−	+ N
16.	*C. glabrata* CBS138	++	+ N	−	−	−	−	−	+ N
17.	*C. glabrata* NGY7	++	+ N	+ N	+ −	−	−	−	+ N
18.	*C. glabrata* NGY14	++	+ N	−	−	−	−	−	−
19.	*P. kudriavzevii* NGY12	++	+ N	+ N	++	−	−	−	+ N
20.	*P. kudriavzevii* NGY13	++	+ N	+ N	+ −	−	−	−	+ N
21.	*P. kudriavzevii* NGY15	++	+ N	+ N	+ −	−	−	−	+ N
22.	*P. kudriavzevii* NGY16	++	+ N	+ N	++	−	−	−	+ N
23.	*P. kudriavzevii* NGY20	++	+ N	+ N	+ −	−	−	−	+ N
24.	*C. dubliniensis* NGY5	++	+ N	+ N	++	−	−	−	+ N
25.	*C. tropicalis* NGY3	++	+ N	+ N	++	−	+ N	−	+ N
26.	*C. tropicalis* NGY4	++	+ N	+ N	++	−	−	+ N	+ N
27.	*C. tropicalis* NGY6	++	+ N	+ N	++	−	−	−	+ N
28.	*C. tropicalis* NGY9	++	+ N	+ N	++	−	−	−	+ N
29.	*C. tropicalis* NGY17	++	+ N	+ N	++	−	−	−	+ N
30.	*C. tropicalis* NGY18	++	+ N	+ N	++	−	−	−	+ N
31.	*C. tropicalis* NGY19	++	+ N	+ N	++	−	+ N	−	+ N
32.	*C. tropicalis* NGY21	++	+ N	+ N	++	−	−	−	+ N
33.	*C. tropicalis* NGY22	++	+ N	+ N	++	−	−	−	+ N
34.	*C. tropicalis* NGY23	++	+ N	+ N	++	−	−	−	+ N
35.	*C. tropicalis* NGY24	++	+ N	+ N	++	−	−	−	+ N
36.	*C. tropicalis* NGY25	++	+ N	+ N	++	−	−	−	+ N

^a^(++): growth and fermentation both positive

^b^(+ N): growth positive but fermentation data not available

^c^(−): no growth and no fermentation

^d^(+−): growth positive but no fermentation. All these experiments were carried out in YNB medium containing different sugars (2.0% w/v)

### Effect of pretreatment-generated inhibitors on yeast growth

**Fig. 2:**
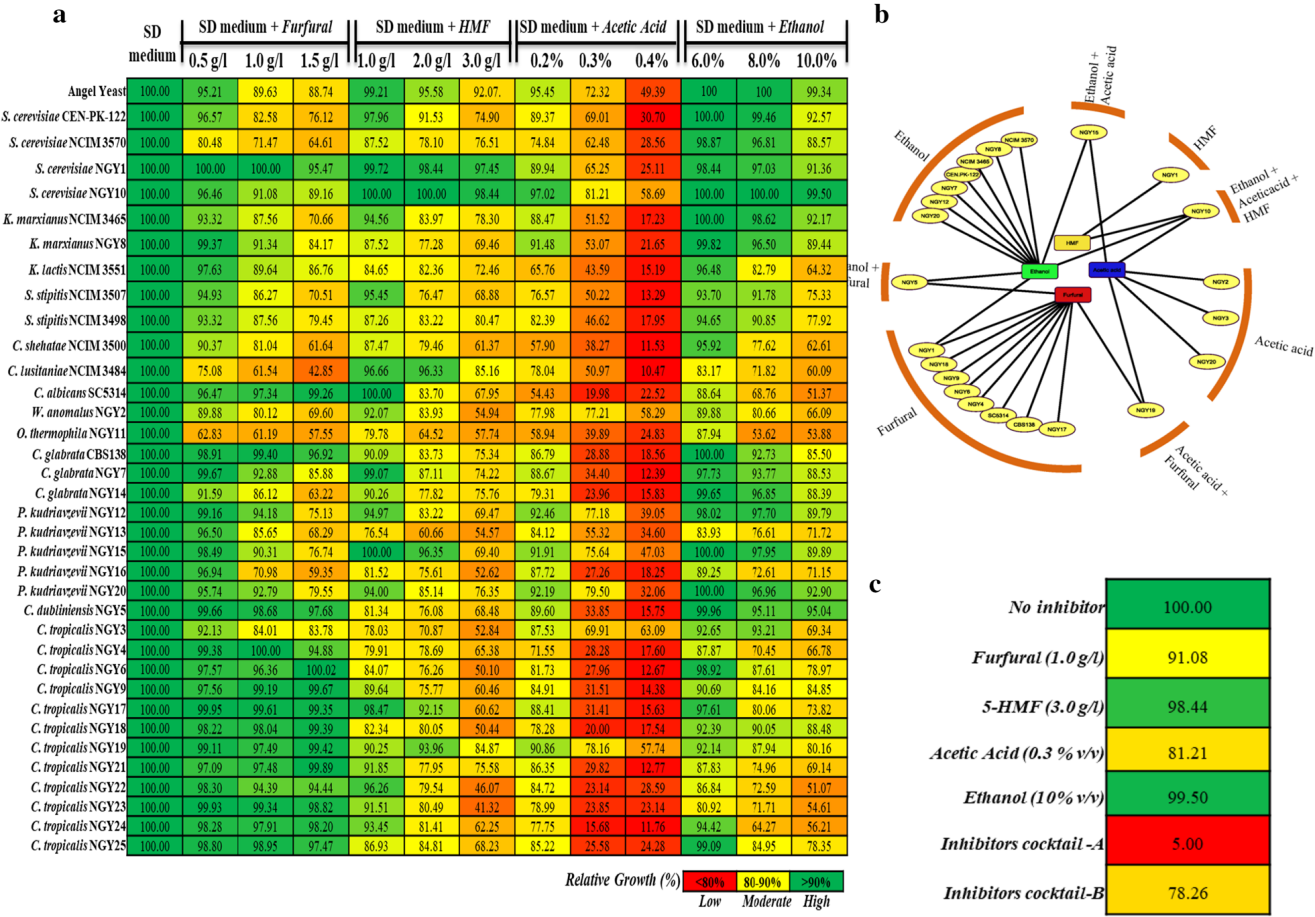
Growth phenotypes in the presence of pretreatment-generated inhibitors and fermentation stresses. **a** The cells were grown in SD medium containing 2.0% glucose with varying concentration of pretreatment-generated inhibitors such as furfural (0.5 g/l, 1.0 g/l and 1.5 g/l), 5-HMF (1.0 g/l, 2.0 g/l and 3.0 g/l), acetic acid (0.2%, 0.3% and 0.4% v/v) and ethanol (6.0%, 8.0% and 10% v/v) at 40 °C. Relative growth in the presence of inhibitors was calculated by considering 100% growth in the absence of inhibitors. **b** Chemogenetic network profile: In silico Chemogenetic network profile was generated using Cytoscape 3.6.0 software using 1.5 g/l of furfural, 3.0 g/l of 5-HMF, 0.3% v/v of acetic acid and 10.0% v/v ethanol individually as well as in combinations. **c** Isolate NGY10 growth phenotypes in the presence of inhibitor: % growth reduction in presence of 1.0 g/l furfural, 3.0 g/l 5-HMF, 0.3% v/v acetic acid, 10% v/v ethanol, cocktail A (1.0 g/l furfural, 3.0 g/l 5-HMF, 0.3% v/v acetic acid and 10% v/v ethanol) and cocktail B (furfural: 0.618 g/l, 5-HMF: 0.748 g/l, acetic acid: 0.18% v/v and ethanol 5.0% v/v) as compared to without inhibitors in SD medium containing 2.0% glucose at 40 °C

LH fermentation is challenging due to stressful conditions such as pretreatment generated inhibitors (furfural, 5-hydroxymethylfurfural and acetic acid), ethanol and elevated temperature. During fermentation, these stresses inhibit the growth of the microbes leading to reduced ethanol yield and productivity. Therefore, yeast isolates with inherent enhanced stress-tolerant phenotypes are very much in demand for industrial lignocellulosic ethanol production. In search of robust yeast isolates, we analyzed the growth inhibition at 40 °C in the presence of inhibitors and relative % reduction in growth was measured by considering 100% growth without inhibitors in glucose-containing SD medium (Fig. 2). Accordingly, the isolates were divided into three categories, least inhibited (< 10% growth reduction; shown in green color), moderately inhibited (10–20% growth reduction; shown in yellow color) and highly inhibited (> 20% growth reduction; shown in red color) (Fig. 2a).

### Effect of furfural on growth

Most of the pre-treatment process generates less than 1.0 g/l of furfural in the lignocellulosic hydrolysate; hence, we tested the growth inhibition in the presence of 0.5–1.5 g/L of furfural (Fig. 2a). Interestingly, at all tested concentrations, isolates of *S. cerevisiae*, *C. glabrata* and *C. tropicalis* displayed the least growth inhibition. None of the tested isolates displayed significant growth inhibition below 0.5 g/l of furfural (data not shown). However, isolates NGY1, SC5314, CBS138, NGY5 and isolates of *C. tropicalis* except NGY3, displayed least growth inhibition at 1.5 g/l of furfural. Additionally, isolates NGY10, NGY8, NGY7, NGY12, NGY15 and NGY20 displayed least growth inhibition at 1.0 g/l of furfural. Together, isolates NGY1, CBS138 and *C. tropicalis* except isolate NGY3 displayed minimal growth inhibition (4.53%, 3.02% and < 6.0% inhibition) at 1.5 g/l of furfural.

### Effect of 5-(hydroxymethyl) furfural on growth

The growth inhibition of yeast isolates was tested in the presence of 1.0–3.0 g/L of 5-HMF (Fig. 2a), as most of the lignocellulosic hydrolysates contain below 2.0 g/l of 5-HMF. None of the tested isolates displayed significant growth inhibition below 0.5 g/l of 5-HMF (data not shown). However, at 3.0 g/l of 5-HMF, isolates NGY1 and NGY10 showed the least growth inhibition. However, isolates NGY1, NGY10, CEN.PK-122, NCIM3484, NGY15, NGY17 and NGY19 displayed least growth inhibition at 2.0 g/l of 5-HMF. Whereas, isolates NCIM3465, NCIM3551, NCIM3498, SC5314, NGY2, CBS138, NGY7, NGY12, NGY20, NGY18, NGY23, NGY24 and NGY25 growth was moderately inhibited at 2.0 g/L of 5-HMF. Together, *S. cerevisiae* isolates NGY1 and NGY10 were most tolerant to 5-HMF (Fig. 2a).

### Effect of acetic acid on growth

Among lignocellulosic hydrolysate inhibitors, acetic acid is considered as one of the most potent inhibitors of growth. The inhibitory effect of acetic acid was tested at graded concentrations (0.2 to 0.4% v/v) (Fig. 2a). Initially, the pH of the medium was 5.5, while it was changed to 4.12, 3.68 and 3.27 after addition of 0.2% v/v, 0.3% v/v and 0.4% v/v acetic acid, respectively. Interestingly, none of the tested isolates displayed growth inhibition below 0.2% v/v (data not shown); however, at 0.4% v/v, more than 40% growth inhibition was observed in all tested isolates. Isolate NGY10 was the only isolate with moderate growth inhibition at 0.3% v/v, and all other isolates showed high growth inhibition. However, at 0.2% v/v of acetic acid isolates NGY10, NGY8, NGY12, NGY15, NGY20 and NGY19 displayed least growth inhibition. Together, isolate NGY10 was the most acetic acid tolerant with minimum growth inhibition (18.79%) at 0.3% v/v (Fig. 2a). Moreover, at similar pH (maintained by HCl), isolate NGY10 displayed a very mild reduction in cell growth, indicating that acetic acid acts as a decoupling agent for yeast cell growth (data not shown). Further, we evaluated the acetic acid-tolerant phenotypes of isolate NGY10 along with the two industrial yeast strains (CEN.PK-122 and Angel yeast) at 30 °C. Interestingly, isolate NGY10 displayed higher tolerance to acetic acid at 30 °C (26.01% growth reduction at 0.4% v/v acetic acid) as compared to 40 °C and relatively more tolerant phenotypes were observed as compared to Angel yeast and CEN.PK-122 at 30 °C (Additional file 1).

### Effect of ethanol on growth

The ethanol-tolerant phenotypes of the yeast isolates were tested at graded concentrations (6.0% to 10.0% v/v) (Fig. 2a). None of the tested isolates displayed growth inhibition at 6.0% v/v of ethanol (data not shown). However, only isolates CEN.PK-122, NGY1, NGY10, NCIM 3465, NGY8, NGY7, NGY5, NGY20 displayed least growth inhibition with 10.0% v/v of ethanol. Additionally, isolates NCIM3570, NCIM3507, NCIM3498, CBS138, NGY7, NGY14, NGY15, NGY3 and NGY18 growth was least inhibited at 8% v/v. Together, isolates NGY10, NGY5, and NGY20 displayed ethanol-tolerant phenotypes with minimal growth inhibition (0.5%, 4.96% and 7.43%, respectively) at 10.0% v/v (Fig. 2a). Further, we evaluated the ethanol-tolerant phenotypes of isolate NGY10 along with the two industrial strains (CEN.PK-122 and Angel yeast) at 30 °C. Interestingly, isolate NGY10 displayed higher tolerance to ethanol at 30 °C (25.8% growth reduction at 16% v/v ethanol) as compared to 40 °C and relatively more ethanol-tolerant phenotypes were observed as compared to Angel yeast and CEN.PK-122 at 30 °C (Additional file 1).

**Fig. 3:**
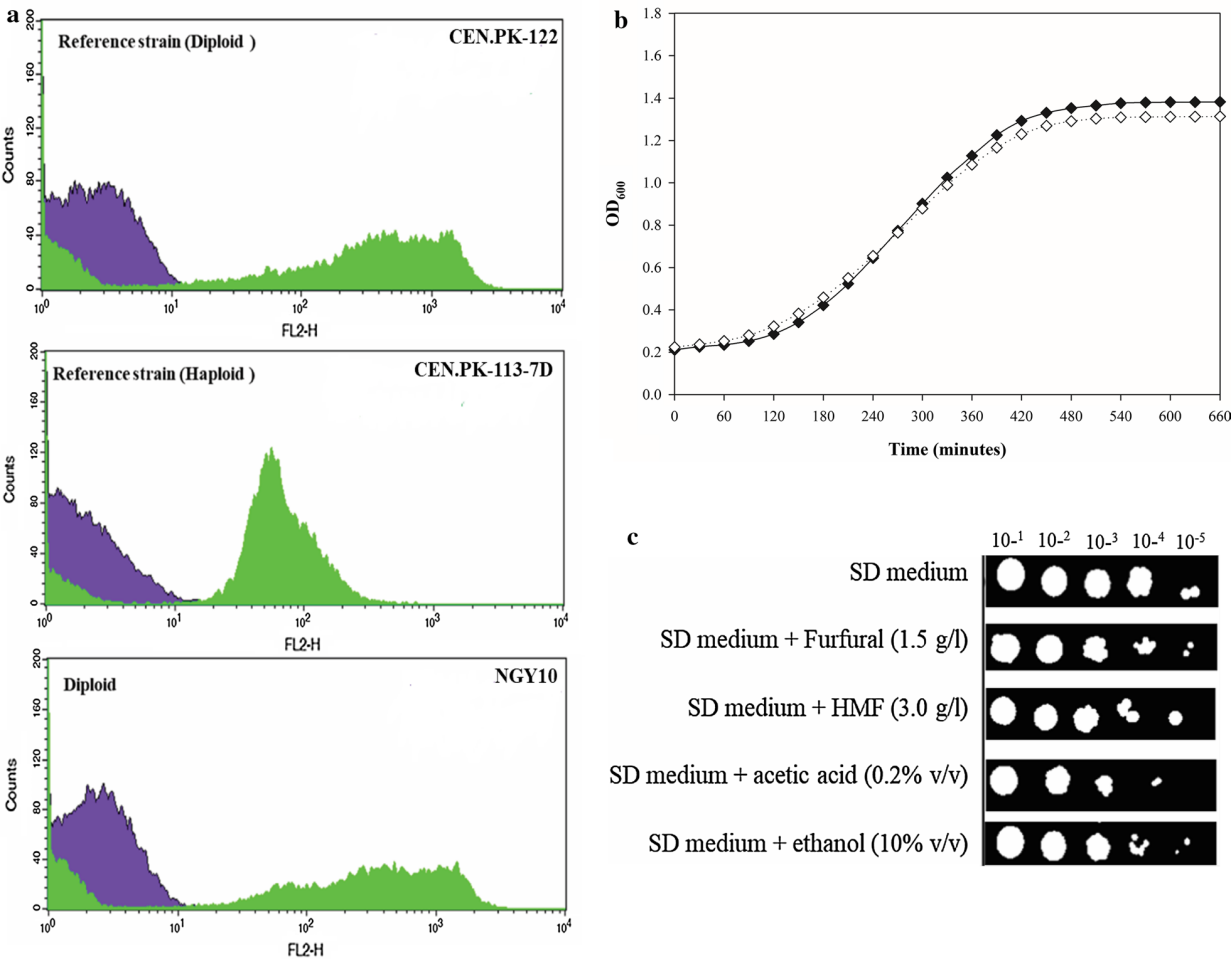
Ploidy determination, thermotolerant phenotypes and pretreatment-generated inhibitors tolerance profile of isolate NGY10. **a** Ploidy determination: Total DNA content of the cells was analyzed by flow cytometry followed by propidium iodide (PI) staining. Ploidy was determined by comparing the FACS spectra of isolate NGY10 with the spectra of reference haploid (CEN.PK-1137D) and diploid (CEN.PK-122) strains. Unstained and stained cells are represented by purple and green colors. **b** Thermotolerant phenotype: isolate NGY10 cells were grown in YEPD broth at 30 °C (filled black diamonds) and 40 °C (clear black diamonds), respectively, followed by OD_600_ measurement after every 30 min interval. **c** Pre-treatment inhibitors-tolerant phenotypes: Serial tenfold dilution of isolate NGY10 cells (OD_600_ = 0.1) was spotted on SD agar plates containing 2.0% glucose and inhibitors (Furfural 1.5 g/l, 5-HMF 3.0 g/l, acetic acid 0.2% v/v and ethanol 10% v/v), and incubated at 40 °C for 24 h

Since, isolate NGY10 displayed stress-tolerant phenotypes with all the tested inhibitors, next we tested the growth inhibition of isolate NGY10 in the presence of a cocktail of inhibitors. Surprisingly, no growth was observed with inhibitor cocktail A (1.0 g/l furfural, 3.0 g/l 5-HMF, 0.3% acetic acid and 10% ethanol), whereas with inhibitors cocktail B (furfural: 0.618 g/l, 5-HMF: 0.748 g/l, acetic acid: 0.18% v/v and ethanol 5.0% v/v) 21.24% growth inhibition was observed (Fig. 2c). More severe growth inhibition in the presence of inhibitor cocktails is a common phenomenon and has been reported earlier [27]. The inhibitor-tolerant phenotype of isolate NGY10 was also confirmed by spotting assay on SD agar plate containing pretreatment-generated inhibitors (Fig. 3c).

The growth inhibition by above-tested inhibitors individually as well as in combinations was also analyzed in silico through Chemogenetic network (Fig. 2b) generated by Cytoscape 3.6.1 software. Chemogenetic network is a graphical representation, which in silico predicts the tolerant growth phenotypes for given concentration of inhibitors as well as their combinations. Chemogenetic network displayed tolerant phenotypes in agreement with the in vivo study for each inhibitor (Fig. 2a). Interestingly, in the presence of combination of all four inhibitors, none of the tested isolates displayed any growth; whereas in the presence of three inhibitors (5-HMF, acetic acid and ethanol), only isolate NGY10 displayed resistant phenotype (Fig. 2b). However, in the presence of two inhibitors, NGY5 displayed ethanol and furfural, NGY15 displayed ethanol and acetic acid and NGY19 displayed furfural- and acetic acid-tolerant phenotypes (Fig. 2b).

### Ploidy determination

The occurrence of diploids and polyploids in yeasts collected from industrial setup and a correlation between DNA content of the cell with stress tolerance and ethanol fermentation performances have been reported in previous studies [25, 40]. Hence, we determined the ploidy of isolates used in this study by propidium iodide (PI) straining. Among *S. cerevisiae* isolates, NGY10 showed DNA content equivalent to diploid (CEN.PK122) (Fig. 3a), while NGY1 and NCIM3570 showed DNA content between haploid and diploid references (CEN.PK-1137D CEN.PK-122), suggesting aneuploidy (Additional files 2 and 3). Notably, all the isolates of *K. marxianus* and *C. glabrata* along with isolates NCIM 3551 and NCIM 3498 showed haploid phenotypes. Whereas, all the isolates of *P. kudriavzevii* and *C. tropicalis* along with the isolates NCIM 3484, SC5314, and NGY2 were turned out as diploid. The DNA content of isolates NGY11, NCIM 3507 and NCIM 3500 was less than diploids, hence considered as aneuploids. As expected, majority of the isolates were diploids (Additional files 2 and 3). The diploid phenotypes could be due to the higher copy number of genes required for survival in harsh environmental conditions at sample collection sites [40, 41].

### Fermentation at elevated temperatures

Rise in temperature during fermentation reduces the efficiency of mesothermal yeast for ethanol production; therefore, fermentation performance at elevated temperatures is considered as an important characteristic for an industrial yeast. Additionally, thermotolerant yeast isolates are required for developing SSF technology to cope up with the thermal miss alliance optima of commercial cellulase activities and fermentation performances [26, 42]. Hence, we evaluated the fermentation performances at 30 °C, 40 °C and 42 °C, respectively. As expected, all isolates fermented glucose, but lesser ethanol yields were obtained at 40 °C and 42 °C as compared to 30 °C (Additional file 3). Among tested isolates NGY1, NGY10, NCIM3465, NGY8, CBS138, NGY7, NGY12, NGY19, NGY20 and the reference strain CEN.PK-122 produced > 41.0 g/l of ethanol with > 91% efficiency at 30 °C (Additional file 3). However, only isolates NGY10, NGY8, NGY7 and NGY20 produced maximum ethanol at 40 °C with a small reduction in the yield (3.86%, 2.35%, 3.91% and 1.57%, respectively) as compared to 30 °C. While, ethanol yield of all the isolates was highly reduced (up to 30%) at 42 °C as compared to 30 °C (Additional file 3). Notably, isolate NGY10 produced maximum ethanol (46.81 ± 3.11 g/l) with the yield of 93.54%, at 40 °C in 24 h.

Next, we evaluated the fermentation performances using xylose as a sole carbon source under micro-aeration condition (in 100 ml serum bottles containing 50 ml of fermentation broth medium), wherein initially the growth is supported by air followed by anaerobic environment [6]. As expected, *S. cerevisiae* isolates did not ferment xylose (Additional file 4). Among other isolates, NCIM3507 and NCIM3498 belonging to *S. stipitis* species and NCIM3500 produced 6.038 ± 0.31 g/l, 6.393 ± 0.27 g/l and 5.821 ± 0.02 g/l ethanol with the yield of 81.32%, 86.10% and 62.76%, respectively, at 30 °C in 24 h, which were substantially reduced by 18.7%, 22.5% and 7.2%, respectively, at 40 °C (Table 4). As reported earlier, xylose-fermenting isolates produced xylitol at 30 °C, which was substantially increased at 40 °C [6]. Among tested isolates, NGY19 produced maximum 1.41 ± 0.073 g/l of xylitol at 30 °C and 8.33 ± 0.21 g/l of xylitol at 40 °C (Additional file 4).

**Table 4:** Fermentation performance of selected yeast isolates at 30 °C and 40 °C in SD media containing glucose/xylose

S. no.	Yeast strains	30 °C						40 °C					
		Ethanol concentration (g/l)	Ethanol yield (g/g)	Ethanol productivity (g/l//h)	Ethanol efficiency (%)	Glycerol concentration (g/l)	Acetic acid concentration (g/l)	Ethanol concentration (g/l)	Ethanol yield (g/g)	Ethanol productivity (g/l/h)	Ethanol efficiency (%)	Glycerol concentration (g/l)	Acetic acid concentration (g/l)
*Initial sugar = glucose (100.0* *g/l)*													
1.	Angel yeast	48.94 ± 1.38^a^	0.489	2.04	95.69	1.72	0.314	45.91 ± 1.45	0.471	1.91.2	92.17	4.11	0.361
2.	*S. cerevisiae* CEN-PK-122	48.79 ± 0.61	0.488	2.032	95.5	2.414	0.624	41.58 ± 1.08	0.43	1.732	84.148	2.722	0.936
3.	*S. cerevisiae* NGY1	47.59 ± 2.34	0.485	1.983	94.91	3.744	0.37	45.65 ± 2.44	0.47	1.902	91.976	4.374	0.464
4.	*S. cerevisiae* NGY10	49.77 ± 0.34*	0.497	2.073	97.397	4.733	0.377	46.81 ± 3.11*	0.478	1.950	93.542	5.206	0.421
5.	*K. marxianus* NCIM 3465	42.19 ± 3.08	0.48	1.757	93.933	3.945	0.476	41.22 ± 2.87	0.45	1.7175	88.062	4.016	0.465
6.	*K. marxianus* NGY8	46.50 ± 0.99	0.473	1.937	92.563	2.379	0.395	42.46 ± 2.00	0.461	1.769	90.215	3.025	0.395
7.	*C. glabrata* CBS138	43.63 ± 2.23	0.472	1.817	92.367	3.647	0.323	42.72 ± 2.55	0.459	1.78	89.823	4.276	0.316
8.	*C. glabrata* NGY7	47.81 ± 2.11	0.48	1.992	93.933	4.705	0.426	46.09 ± 1.44	0.46	1.92	90.02	3.406	0.447
9.	*P. kudriavzevii* NGY12	45.63 ± 2.71	0.469	1.901	91.78	2.364	0.339	43.60 ± 1.88	0.455	1.816	89.04	2.064	0.149
10.	*P. kudriavzevii* NGY20	43.44 ± 1.38	0.47	1.81	91.976	2.161	0.317	42.12 ± 1.45	0.462	1.755	90.41	2.173	0.344
11.	*C. tropicalis* NGY19	41.49 ± 1.96	0.43	1.81	84.15	2.161	0.317	41.77 ± 1.98	0.429	1.740	84.0	0.918	0.447
*Initial sugar = xylose (50.0* *g/l)*													
12.	*S. stipitis* NCIM3507	6.038 ± 0.31	0.416	0.251	81.321	0.037	0.045	3.37 ± 0.128	0.32	0.14	62.63	0.037	0.045
13.	*S. stipitis* NCIM3498	6.393 ± 0.27	0.439	0.266	86.1	0.021	0.05	3.75 ± 0.133	0.325	0.156	63.625	0.021	0.05
14.	*K. marxianus* NGY8	1.525 ± 0.013	0.359	0.063	70.385	0.048	0.033	1.48 ± 0.025	0.349	0.061	68.31	0.048	0.033
15.	*C. shehatae* NCIM 3500	5.821 ± 0.02	0.320	0.242	62.765	1.11	0.311	3.74 ± 0.016	0.284	0.155	55.58	1.11	0.311
16.	*C. lusitaniae* NCIM 3484	1.509 ± 0.01	0.444	0.062	86.930	0.005	0.082	0.601 ± 0.023	0.430	0.025	84.189	0.005	0.082
17.	*W. anomalus* NGY2	0.704 ± 0.03	0.159	0.029	31.155	0.028	0.046	0.614 ± 0.019	0.138	0.025	27.172	0.028	0.046
18.	*O. thermophila* NGY11	0.445 ± 0.03	0.089	0.018	17.564	0.002	0.17	0.312 ± 0.013	0.021	0.013	4.11	0.002	0.17
19.	*C. glabrata* NGY14	0.11 ± 0.006	0.141	0.005	27.597	0.002	0.03	0.06 ± 0.005	0.005	0.0025	0.98	0.003	0.01
20.	*P. kudriavzevii* NGY12	0.544 ± 0.025	0.405	0.023	79.446	0.001	0.01	0.444 ± 0.025	0.189	0.018	37.131	0.001	0.01
21.	*C. dubliniensis* NGY5	0.535 ± 0.03	0.137	0.022	26.914	0.093	0.09	0.211 ± 0.004	0.003	0.008	0.0	0.093	0.09
22.	*C. tropicalis* NGY22	0.794 ± 0.03	0.235	0.033	46.120	0.12	0.071	0.541 ± 0.028	0.228	0.022	44.690	0.12	0.071

^a^Mean ± standard deviation, *n* = 3; Fermentation volume: 50 ml; pH-5.4; inoculums 5.0% v/v ≈ 1.0 × 10^7^ cells/ml, fermentation time 24 h

* Statistical student t-test of isolate NGY10 with reference strains CEN-PK-122 and Angel yeast for glucose fermentation at 30 °C and 40 °C was performed, and show significance, i.e. *p* < 0.05

Since, none of the tested isolates produced significant amount of ethanol with xylose as a carbon source, for further studies involving SHF and SSF processes, we selected only glucose-fermenting isolates with more than 84% ethanol yield at 40 °C (Table 4).

### Fermentation of rice straw hydrolysates via SHF

Direct fermentation of LH is challenging due to the presence of a cocktail of inhibitors generated during pretreatment and hence in most of the cases, low ethanol yields and productivities have been reported [11, 26]. Additionally, optimum enzymatic hydrolysis occurs at 50 °C; hence, cooling to 30 °C for obtaining maximum ethanol production yield by yeast is another cost-ineffective step in lignocellulosic ethanol production. Hence, yeast isolates with optimum ethanol yield and productivities in LH at 40 °C are desirable. We assessed the fermentation performances of above-selected yeast isolates via SHF using acid- and alkali-pretreated rice straw enzymatic hydrolysates (APRSEH-1 and APRSEH-2, respectively) at 40 °C under batch cultures in shake flasks (as described in "Methods"). During SHF, no filtration, centrifugation, autoclaving and vessel change was performed. APRSEH-1 slurry contained 33.76 g/l of sugar (glucose: 26.38 g/l and xylose: 7.38 g/l), furfural: 0.618 g/l, 5-HMF: 0.748 g/l and acetic acid: 1.91 g/l, whereas APRSEH-2 slurry contains 22.78 g/l sugar (glucose: 17.15 g/l and xylose: 5.63 g/l), furfural: 0.142 g/l, 5-HMF: 0.148 g/l and acetic acid: 0.51 g/l. All the tested isolates produced ethanol in the range of 9.45 ± 0.16 g/l to 12.67 ± 0.09 g/l and 5.67 ± 0.13 g/l to 7.18 ± 0.04 g/l with APRSEH-1 and APRSEH-2, respectively (Table 5). Interestingly, isolate NGY10 produced maximum (12.25 ± 0.09 g/l) ethanol with the yield of 92.81% in APRSEH-1 and 7.18 ± 0.04 g/l ethanol with the yield of 91.58% in APRSEH-2. However, isolates NGY1, NGY8, NGY19 and NGY20 produced 11.75 ± 315, 0.13 g/l, 11.55 ± 0.08 g/l, 10.48 ± 0.11 g/l and 10.51 ± 0.17 g/l ethanol with the yield of 87.16%, 316 85.68%, 89.35% and 90.98%, respectively, with APRSEH-1 as substrate. When APRSEH-2 was used as a substrate, isolates NGY1, NGY7, NGY8 and NGY12 produced 7.09 ± 0.09 g/l, 7.02 ± 0.06 g/l, 6.98 ± 0.07 g/l and 318 6.95 ± 0.11 g/l ethanol with the yield of 88.85%, 84.14%, 80.23% and 80.23%, respectively (Table 5). Although several tested isolates fermented APRSEH-1 and APRSEH-2 at 40 °C via SHF, isolate NGY10 produced maximum ethanol yield and productivity (Table 5). Interestingly, isolate NGY10 also produced maximum ethanol yield at 40 °C with glucose as carbon source and displayed least growth inhibition with pretreatment inhibitors, therefore isolate NGY10 was selected for further kinetic studies.

**Table 5:** Fermentation profile of selected glucose fermenting yeast isolates with acid- and alkali-pretreated rice straw enzymatic hydrolysates at 40 °C in 24 h

S. no.	Yeast strains	Acid pre-treated rice straw enzymatic hydrolysate (APRSEH-1)^b^						Alkali pre-treated rice straw enzymatic hydrolysate (APRSEH-2)^c^					
		Ethanol concentration (g/l)	Ethanol yield (g/g)	Ethanol Productivity (g/l//h)	Ethanol efficiency (%)	Glycerol concentration (g/l)	Acetic acid concentration (g/l)	Ethanol concentration (g/l)	Ethanol yield (g/g)	Ethanol productivity (g/l/h)	Ethanol efficiency (%)	Glycerol concentration (g/l)	Acetic acid concentration (g/l)
1.	Angel yeast	11.79 ± 0.08^a^	0.451	0.491	88.258	0.47 ± 0.03	0.52 ± 0.02	7.02 ± 0.02	0.456	0.293	89.24	0.46 ± 0.002	0.73 ± 0.02
2.	*S. cerevisiae* CEN-PK-122	11.65 ± 0.21	0.441	0.485	86.423	1.38 ± 0.07	0.78 ± 0.03	6.95 ± 0.07	0.41	0.289	80.23	1.56 ± 0.06	0.79 ± 0.03
3.	*S. cerevisiae* NGY1	11.75 ± 0.13	0.445	0.489	87.165	0.6 ± 0.02	0.54 ± 0.02	7.09 ± 0.09	0.454	0.295	88.85	0.39 ± 0.012	0.24 ± 0.01
4.	*S. cerevisiae* NGY10	12.25 ± 0.09*	0.474	0.51	92.812	0.54 ± 0.02	0.47 ± 0.011	7.18 ± 0.04*	0.468	0.298	91.58	0.33 ± 0.005	0.18 ± 0.002
5.	*K. marxianus* NCIM 3465	10.89 ± 0.16	0.412	0.453	80.785	0.85 ± 0.04	0.66 ± 0.02	6.19 ± 0.03	0.361	0.257	70.64	0.64 ± 0.021	0.36 ± 0.002
6.	*K. marxianus* NGY8	11.55 ± 0.08	0.437	0.481	85.681	0.37 ± 0.01	1.87 ± 0.07	6.98 ± 0.07	0.41	0.29	80.23	0.16 ± 0.003	1.57 ± 0.008
7.	*C. glabrata* CBS138	10.17 ± 0.07	0.419	0.423	82.003	0.28 ± 0.03	0.74 ± 0.032	5.67 ± 0.13	0.331	0.236	64.77	0.07 ± 0.002	0.45 ± 0.01
8.	*C. glabrata* NGY7	10.92 ± 0.13	0.436	0.455	85.513	0.34 ± 0.02	0.81 ± 0.03	7.02 ± 0.06	0.43	0.292	84.14	0.13 ± 0.003	0.52 ± 0.002
9.	*P. kudriavzevii* NGY12	9.45 ± 0.16	0.425	0.393	83.264	0.34 ± 0.01	0.67 ± 0.03	6.95 ± 0.11	0.41	0.289	80.23	0.13 ± 0.001	0.39 ± 0.003
11.	*P. kudriavzevii* NGY20	10.51 ± 0.17	0.456	0.437	89.346	0.34 ± 0.02	0.67 ± 0.033	5.71 ± 0.12	0.332	0.237	64.97	0.13 ± 0.001	0.38 ± 0.002
12.	*C. tropicalis* NGY19	10.48 ± 0.11	0.464	0.436	90.988	0.47 ± 0.03	0.46 ± 0.021	4.98 ± 0.08	0.29	0.207	56.75	0.26 ± 0.011	0.04 ± 0.001

^a^Mean ± standard deviation for *n* = 3; Fermentation pH-5.4; Initial sugar = 26.38 g/l, inoculums 5.0% v/v ≈ 1.0 × 10^7^ cells/ml, fermentation time 24 h

^b^APRSEH-1 consisted of sugar 33.76 g/l (glucose: 26.38 g/l and xylose: 7.38 g/l), furfural: 0.618 g/l, HMF: 0.748 g/l and acetic acid: 1.91 g/l

^c^APRSEH-2 consisted of sugar 22.78 g/l (glucose: 17.15 g/l and xylose: 5.63 g/l), furfural: 0.142 g/l, HMF:0.148 g/l and acetic acid:0.51 g/l

* Statistical student t-test of isolate NGY10 with reference strains CEN-PK-122 and Angel yeast for glucose fermentation of APRSEH-1 and APRSEH-2 was performed, and show significance, i.e. *p* < 0.05

### Kinetics of ethanol production in SHF

**Fig. 4:**
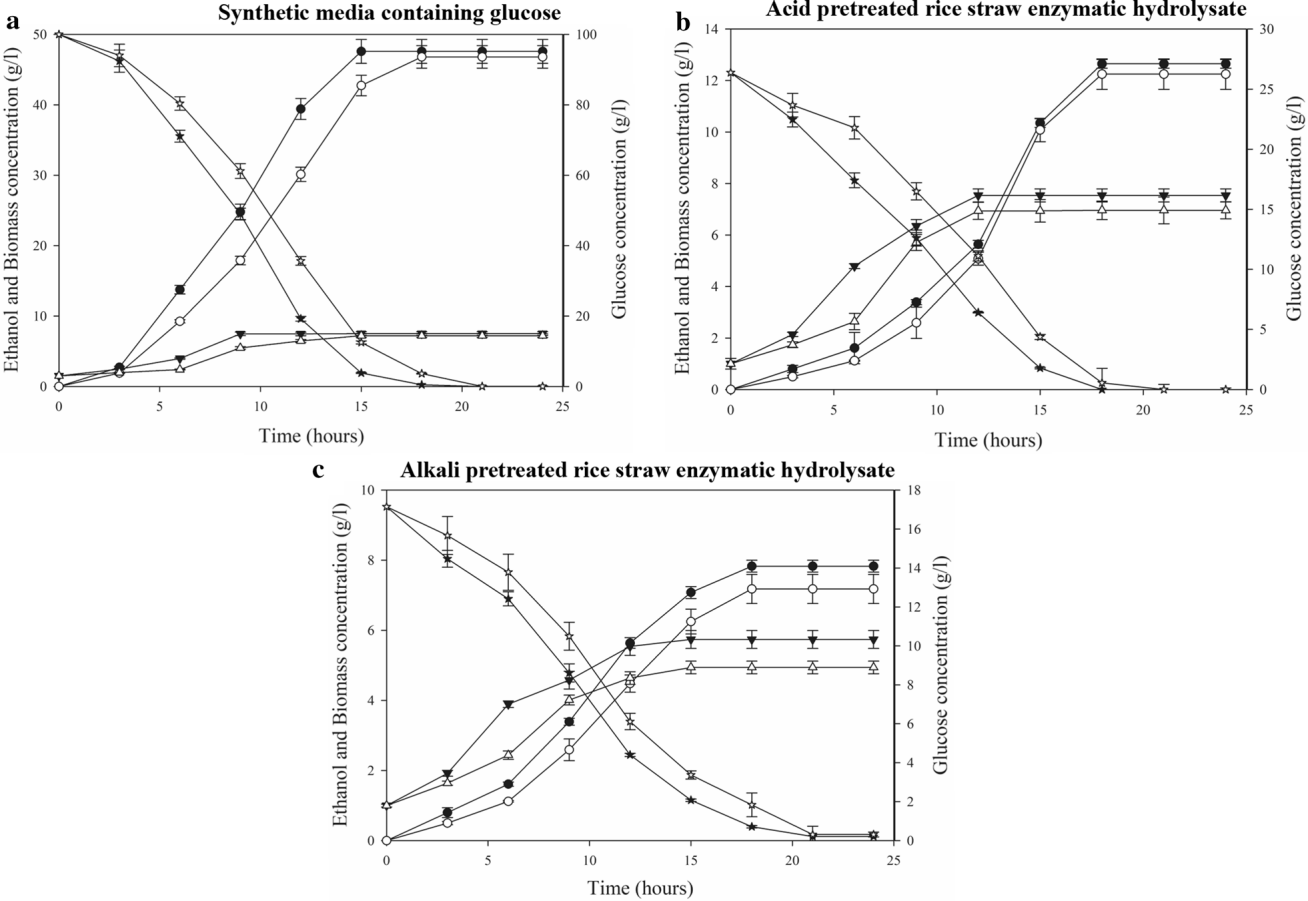
Fermentation kinetics: Isolate NGY10 cells were grown in YEPD broth and 5% v/v inoculum of overnight grown cells was diluted in different fermentation media and fermentation was performed for 24 h. Glucose (circle), ethanol (triangle) and cell biomass (star) were estimated at 30 °C (filled symbols) and 40 °C (clear symbols) after every 3.0 h. **a** Fermentation kinetics in synthetic media containing 100 g/l glucose. **b** Fermentation kinetics in acid-pretreated rice straw enzymatic hydrolysate (containing glucose: 26.38 g/l; xylose: 7.38 g/l; furfural: 0.618 g/l; HMF: 0.748 g/l and acetic acid: 1.91 g/l) and **c** Fermentation kinetics in alkali pre-treated rice straw enzymatic hydrolysate (containing glucose: 17.15 g/l; xylose: 5.63 g/l; furfural: 0.142 g/l; HMF:0.148 g/l and acetic acid:0.51 g/l)

Kinetic studies of ethanol production were performed with each glucose, APRSEH-1 and APRSEH-2 at 30 °C and 40 °C, respectively, by employing isolate NGY10 (Fig. 4; Table 6). During this study, three kinetic parameters including the rate of substrate utilization (*Q*_S_), the rate of biomass production (*Q*_X_) and the rate of product formation (*Q*_P_) were analyzed. As expected, *Q*_S_, *Q*_X_ and *Q*_P_ were lower at 40 °C as compared to 30 °C. As expected at 40 °C *Q*_S_, *Q*_X_ and *Q*_P_ were lower as compared to 30 °C. In the presence of glucose *Q*_P_ was 3.17 g/l/h and *Q*_S_, *Q*_X_ were 6.41 g/l/h and 0.50 g/l/h at 30 °C, which were reduced to 2.6 g/l/h, 5.353 g/l/h and 0.40 g/l/h, respectively, at 40 °C. Similarly, with APRESH-1 and APRESH-2, the *Q*_P_ was 0.703 g/l/h and 0.435 g/l/h at 30 °C, and the small reduction was observed at 40 °C (Table 6). The reduction in *Q*_P_, *Q*_S_ and *Q*_X_ at 40 °C could be due to the slightly longer lag phase as compared to 30 °C (Fig. 4) [6, 25].

**Table 6:** Fermentation kinetics of *S. cerevisiae* NGY10 in various fermentation media

S. no.	Kinetic parameters	Synthetic media containing glucose		Acid-pretreated rice straw hydrolysate		Alkali-pretreated rice straw hydrolysate	
		30 °C	40 °C	30 °C	40 °C	30 °C	40 °C
1.	Initial sugar (*S*_t_) (g/l)	100	100	26.38	26.38	17.15	17.15
2.	Residual sugar (*S*_t_) (g/l)	3.82	3.64	0.433	0.551	0.71	1.832
3.	Sugar consumption rate (*Q*_S_) (g/l/h)	6.412	5.353	1.47	1.22	0.913	0.765
4.	Sugar consumed (%)	96.18	96.36	100	99.449	99.29	98.167
5.	Fermentation time (h)	15	18	18	18	18	18
6.	Maximum ethanol concentration (g/l)	47.59	46.81	12.65	12.25	7.83	7.18
7.	Ethanol yield coefficient, *Y*p/s (g/g)	0.495	0.485	0.479	0.474	0.476	0.468
8.	Ethanol production rate (*Q*_P_) (g/l/h)	3.173	2.6	0.703	0.680	0.435	0.398
9.	Fermentation efficiency (%)	96.83	95.06	93.84	92.81	93.204	91.728
11.	Cell biomass concentration (g/l)	7.51	7.21	7.54	6.96	5.74	4.94
12.	Cell biomass production rate, (*Q*_X_) (g/l/h)	0.5	0.40	0.418	0.386	0.318	0.274
13.	Specific growth rate (*μ*) (h^−1^)	0.226	0.21	0.18	0.161	0.178	0.155

### Fermentation in presence of high sugar concentration

**Fig. 5:**
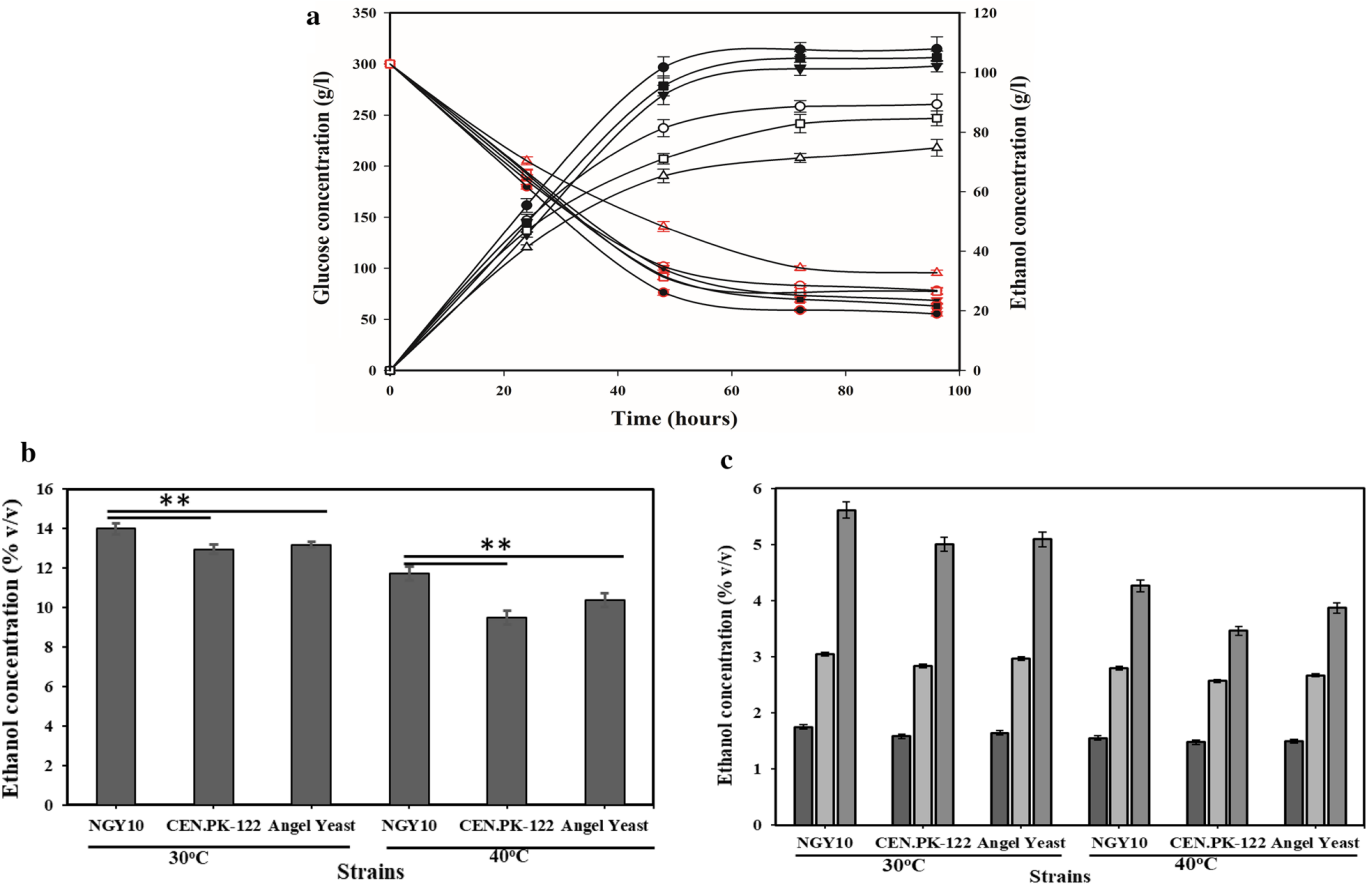
Fermentation profile in the presence of high sugar concentration. **a** Fermentation kinetics of isolate NGY10 (circle), CEN.PK-122 (triangle) and Angel yeast (square) in presence of 30% w/v glucose at 30 °C (filled symbols) and 40 °C (clear symbols), respectively. Ethanol and glucose concentrations are represented by black and red edges symbols, respectively. **b** Comparative ethanol yield with 30% w/v glucose. **c** Comparative ethanol yield with ×1 (black coloured bar), ×2 (light grey coloured bar) and ×4 (dark grey coloured bar) concentrated acid-pretreated rice straw hydrolysate. Statistical Student t-test for ethanol yield was performed for isolate NGY10 with reference strains CEN-PK-122 and Angel yeast, and showed significance (*p* < 0.05)

To access the potential of isolate NGY10 for ethanol production, we evaluated the fermentation performances in the presence of high sugar concentrations at 30 °C and 40 °C, respectively. The fermentation performance of isolate NGY10 was compared with two known industrial strains (CEN.PK-122 and Angel Yeast) in the presence of 30% w/v glucose as well as in 2× and 4× concentrated acid-pretreated rice straw hydrolysates. Interestingly, at 30 °C, isolate NGY10 produced maximum 110.38 ± 3.27 g/l (13.99% v/v) ethanol with the yield of 86.3%, which was reduced to 92.31 ± 3.39 g/l (11.7% v/v) with the yield of 81.49% at 40 °C in the presence of 30% w/v glucose. However, CEN.PK-122 and Angel yeast produced 102.14 ± 1.88 g/l (12.94% v/v) and 103.9 ± 2.14 g/l (13.18% v/v) ethanol with the yield of 81.24% and 83.6, respectively, at 30 °C (Fig. 5a, b), which were reduced to 74.76 ± 2.84 g/l (9.47% v/v) and 81.98 ± 248 g/l (10.39% v/v) with the yields of 71.52% and 74.38% for CEN.PK-122 and Angel Yeast, respectively, at 40 °C. Notably, in the presence of 4× concentrated RS hydrolysate (initial glucose 96.34 g/l ± 2.21) isolate NGY10 produced maximum 44.32 ± 0.82 g/l (5.67% v/v) ethanol with the yield of 81.34% at 30 °C, which was reduced to 33.66 ± 1.04 g/l (4.26% v/v) with the yield of 73.87% at 40 °C (Fig. 5c). As expected, all the tested yeast isolates produced lower ethanol yields at 40 °C as compared to 30 °C. However, a minimum reduction in ethanol yield at 40 °C was observed in case of isolate NGY10 (4.81% with glucose and 7.47% RS hydrolysate), whereas higher reduction in ethanol yields was observed for CEN.PK-122 (9.72% with glucose and 13.71% with RS hydrolyzate) and Angel yeast (9.22% with glucose and 11.47% with RS hydrolysate) at 40 °C. Although 12–16% ethanol titer were reported in previous studies at 30 °C using 30%–35% initial sugar and modified strains such as CEN.M1 [43] and S288C [44]. None of the studies reported comparable ethanol titre at 40 °C. Taken together, these results suggested that isolate NGY10 is a promising candidate for industrial ethanol production.

### Fermentation of rice straw hydrolysate via SSF

**Fig. 6:**
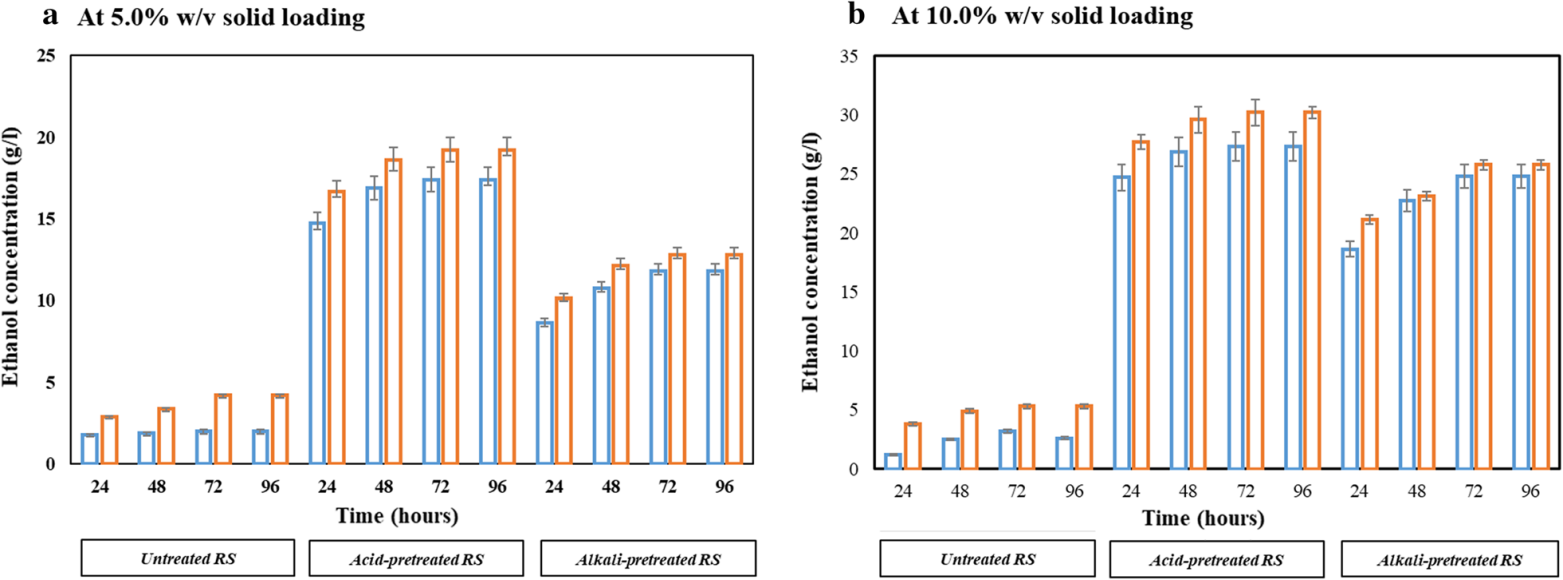
Acid- and alkali-pretreated RS fermentation via SSF without pre-saccharification (blue coloured bar) and with pre-saccharification (orange coloured bar), using isolate NGY10. SSF was performed at 40 °C for 72 h employing 15 FPU cellulase/g of RS and 5.0% v/v inoculums of overnight YEPD grown isolate NGY10. **a** With 5.0% w/v solid loading and **b** with 10.0% w/v solid loading. For SSF without pre-saccharification both cellulase and inoculums were added simultaneously; however, in case of pre-saccharification, cellulases were added to RS and incubated at 50 °C for 6 h before adding the yeast inoculums

Fermentation potential of isolate NGY10 in SSF (with and without pre-saccharification) was evaluated at 40 °C using untreated, acid-pretreated and alkali-pretreated RS for 72 h. During SSF, 5.0% w/v and 10.0% w/v solid loading of pretreated RS with 15 FPU of cellulase/g of dry biomass was used. Since cellulase exhibit maximum activity at 50 °C; hence, pre-saccharification at 50 °C for 6 h was performed in parallel before adding the yeast cells. As reported earlier, we expected that pre-saccharification will enhance the ethanol yield [8, 26, 45]. Without pre-saccharification, 5.0% w/v solid loading of untreated, acid- and alkali-pretreated RS produced 2.02 g/l, 17.36 g/l and 11.78 g/l ethanol, respectively. Whereas, ethanol production was enhanced to 4.21 g/l, 19.22 g/l and 12.77 g/l with pre-saccharification of untreated, acid-pretreated and alkali-pretreated RS, respectively (Fig. 6a). Interestingly, at 10% w/v solid loading higher ethanol was produced (Fig. 6b). Without pre-saccharification, maximum ethanol production was 3.2 g/l, 27.36 g/l and 24.78 g/l; whereas, with pre-saccharification, 5.3 g/l, 30.22 g/l and 25.77 g/l ethanol were produced by untreated, acid-pretreated and alkali-pretreated RS, respectively (Fig. 6b). Notably, acid-pretreated RS with pre-saccharification produced maximum ethanol of 30.22 g/l with the efficiency of 86.43% in SSF. To the best of our knowledge, it was higher than the other recently published reports involving SSF processes, including 70.7% [46], 56.3% [47] and 80.65% [26] by employing *S. cerevisiae* isolates and 77.7% by employing *K. marxianus* isolate [48].

## Discussion

Lignocellulosic ethanol production is challenging due to the presence of pretreatment generated inhibitors in the enzymatic hydrolysates and reduced fermentation performances at elevated temperature. Use of thermotolerant yeast isolates will reduce the cooling cost and contamination during fermentation, and are potential candidates for SSF. Although in recent years several thermotolerant and inhibitors-tolerant yeast isolates have been isolated, but their fermentation performances are still below par [6, 25, 26, 42]; therefore, the search for robust yeast isolates is still relevant. To untap the potential of yeast isolates, isolated from natural habitats related to lignocellulosic ethanol fermentation, 36 yeast isolates with minimal growth inhibition at 40 °C as compared to 30 °C were selected. These isolates belonged to six genera including *Saccharomyces*, *Kluyveromyces, Scheffersomyces, Candia, Ogatea* and *Wickerhamomyces* (Table 1). Among them, isolates NGY7, NGY8, NGY10, NGY20 and NCIM3465 displayed less than 5.0% increase in *T*_d_ at 40 °C as compared to 30 °C. Interestingly, a distillery waste isolate NGY10 belonging to *S. cerevisiae* species displayed minimal (3.84%) increase in *T*_d_ at 40 °C. The increase in *T*_d_ leads to a slight delay in the stationary phase (Table 2; Fig. 3b), which could be due to the down-regulation of growth- and metabolism-associated genes [6, 49, 50].

Inhibitors generated during pretreatment (Furfural, 5-HMF, Acetic acid, etc.) inhibit yeast growth. Furfural reduces growth by increasing lag phase by inhibiting glycolysis, Krebs’s cycle, dehydrogenases activity and inducing oxidative stress [51], whereas 5-HMF inhibits glycolysis and dehydrogenases [52, 53]. Generally, *C. tropicalis* isolates are considered comparatively more furfural resistant than *S. cerevisiae* [6]. In agreement with this, in this study, *C. tropicalis* isolates displayed less growth inhibition as compared to *S. cerevisiae* in the presence of furfural. Recently, industrial yeast *S. cerevisiae* Fm17 and *S. cerevisiae* 27P displayed 11% and 12% growth inhibition in the presence of 1.38 g/l of furfural [27], and *S. cerevisiae* JRC6 showed 28% growth reduction at 1.0 g/l of furfural [6]. *S. cerevisiae* Fm17 and *S. cerevisiae* 27P also displayed 22% and 27% growth inhibition in the presence of 2.81 g/l of 5-HMF [27], and *S. cerevisiae* JRC6 showed about 40% reduction in growth at 2.0 g/l of 5-HMF. Interestingly, isolates NGY1 and NGY10 displayed only 0.28% and 10.84% growth reduction at 1.5 g/l furfural and 2.55% and 1.56% growth inhibition in the presence of 3.0 g/l of 5-HMF, respectively. These results indicated that at similar concentration furfural is more toxic than 5-HMF. These results were consistent with another published report, wherein more growth reduction was reported with furfural [51]. The growth inhibition in the presence of furfural and 5-HMF is not genus or species specific; it may vary from strain to strain [6].

It was observed that acetic acid inhibits cell growth at lower concentrations and most of the tested isolates displayed significant growth reduction at 0.2% v/v of acetic acid. The growth inhibition of acetic acid was linked with cellular stress generated through ATP consumption during efflux of H^+^ ions [18, 54]. Isolates NGY10 and NGY20 displayed minimal growth reduction (18.79% and 20.5%, respectively) at 0.3% v/v (Fig. 2a), which was comparable to the recently reported 11% growth reduction of industrial yeast *S. cerevisiae* 27P at 3.6 g/l [27]. Although, some previous studies reported higher acetic acid-tolerant phenotypes for some *S. cerevisiae* isolates, such as Fm17 (7.2 g/l) [27], Ethanol Red (5.6 g/l) [55] and B44 (0.6% v/v) [56], but these were performed at 30 °C. In case of ethanol, isolates NGY10, NGY5 and NGY20 were tolerant to 10% v/v ethanol and showed least growth inhibition (0.5%, 4.96% and 7.43%, respectively). Notably, the ethanol tolerance of isolate NGY10 was higher than the reference strains CEN.PK-122 and Angel yeast at 40 °C (Fig. 2a). Interestingly, 0.5% growth inhibition of isolate NGY10 in the presence of 10% ethanol was lower than the previously reported values for *S. cerevisiae* isolates such as DBTIOC S24 [26], PDR18 mutant [57], SPT15 mutant [58] and UVNR56 [59]. Although, yeast isolates with more ethanol tolerance than isolate NGY10 were reported in previous studies, but all these studies were performed at 30 °C [60–62]. It is has been reported that ethanol inhibits the cell growth by mitochondrial DNA damage, hexokinase and dehydrogenases inactivation and altered cellular lipids/fatty acids composition [63, 64].

Although, yeast isolates NGY7, NGY8 and NGY20 displayed slight growth reduction in the presence of pretreatment-generated inhibitors, but high fermentation performances at 40 °C in the presence of inhibitors are critical for designing SSF and SScF. Interestingly, a sugarcane distillery waste isolate NGY10 and a dairy waste isolate NGY8 displayed almost comparable fermentation yields at 30 °C and 40 °C using glucose as carbon source (Table 4). Notably, all tested isolates were poor in xylose fermentation at 30 °C and ethanol yields were further reduced at 40 °C (Table 4), as expected xylose fermenting isolates produced a significant amount of xylitol (Additional file 4). In agreement to the fermentation performances, the growth of isolates NGY10 and NGY8 was least inhibited at 40 °C (Table 2). Isolate NGY10 also displayed high fermentation potential in SHF using APRSEH-1 and APRSEH-2 and produced 92.81% and 91.58% ethanol yields at 40 °C in the presence of inhibitor generated during pretreatment (Tables 4, 5). The above-produced ethanol yields were either higher or equivalent to the recently published reports, wherein industrial yeast isolates were tested for LH fermentation. A distillery spent isolate produced 83.73% efficiency with acid pre-treated RS hydrolysate at 42 °C [26], a distillery waste isolate produced 87.9% efficiency with alkali-pretreated RS at 40 °C [6] and another distillery waste isolate produced 94% efficiency with hydrothermal *Eucalyptus* wood hydrolysate [28]. Notably, when isolate NGY10 was tested with 30% glucose and 4× concentrated RS hydrolysate as carbon sources, we achieved 86.3% and 81.34% fermentation efficiency at 30 °C (Fig. 5c). Although higher ethanol titers (12–16%) were achieved in the previous studies using CEN.M1 [43] and S288C [44] strains, but as per our knowledge, none of these isolates produced equivalent ethanol to isolate NGY10 at 40 °C.

Traditionally, lignocellulosic ethanol production is a multistep process involving pretreatment and enzymatic saccharification and fermentation. Optimum pretreatment and saccharification efficiencies were achieved at high temperatures, while fermentation at a lower temperature. Each temperature requires a separate process step, which increases the cost of ethanol production. Therefore, combining these processes in SSF is considered as an important step towards developing a cost-effective LH fermentation technology [26, 65]. One of the pre-requisites of successful SSF is achieving high ethanol production and yield at 40 °C using LH. Isolate NGY10 displayed remarkable ethanol production and yield at 40 °C with glucose in SHF. Interestingly, with acid-pretreated RS at 10% w/v solid loading and 6 h of pre-saccharification (50 °C), 30.22 g/l of ethanol with the yield of 86.43% was produced by isolate NGY10 at 40 °C. The obtained ethanol yield in SSF was higher than the recently reported ethanol yields produced by employing *S. cerevisiae* and *K. marxianus* isolates [26, 46–48]. In agreement with the previous studies, a correlation among DNA content, fermentation performances and stress tolerance was observed, and isolate NGY10 was turned out to be diploid with 2n DNA content. Together, isolate NGY10 displayed high potential for lignocellulosic ethanol production through SHF/SSF process and can be considered as a good candidate for developing Simultaneous saccharification and co-fermentation (SScF) and consolidated bioprocessing (CBP) strategies.

## Conclusion

A sugar cane distillery waste isolate NGY10 promised high potential for lignocellulosic ethanol production and developing SScF and CBP strategies. Isolate NGY10 displayed thermotolerant (40 °C), pre-treatment inhibitor and fermentation stress-tolerant phenotypes (1.5 g/l furfural, 3.0 g/l 5-HMF, 0.3% v/v acetic acid and 10.0% v/v ethanol). This isolate also displayed 92.81% and 86.43% fermentation efficiency during SHF and SSF process using dilute acid-pretreated rice straw (RS) at 40 °C. To the best of our knowledge, this is the first study to analyze the fermentation stress- and pretreatment-generated inhibitor-tolerant phenotypes of a broad range of yeast genera in one study simultaneously. The robust yeast isolates (*K. marxianus*, *O. thermophila, C. tropicalis, C. glabrata* and *P. kudreverzii)* identified in this study could be used to produce value-added compounds (xylitol, other sugar alcohols, inulase, etc.) using the lignocellulosic-based material as feedstock.

## Methods

### Media and other chemicals

Yeast extract, peptone and yeast nitrogen base (YNB) without amino acids were procured from BD Difco™ (USA). HiCrome™ differential agar, furfural (99.0%), 5-HMF (97.0%), acetic acid (99.7%), d-glucose and d-xylose were procured from Himedia laboratory, India. Mannose, galactose, l-arabinose, cellobiose, maltose and lactose were procured from Sigma Aldrich, India. All media components and chemicals used in this study were of analytical grade.

### Isolation, procurement and maintenance of yeast

Samples were collected from natural habitats, including distillery wastes, sewage and algal bloom and Dairy wastes (Table 1) in summer (June 2016), when the temperature of collection sites was touching 40 °C–45 °C. The samples were mixed with yeast extract peptone dextrose (YEPD) broth (yeast extract: 10.0 g/l, peptone: 20.0 g/l and glucose: 20.0 g/l) containing antibiotics (chloramphenicol: 0.2 g/l and kanamycin: 30 mg/l), and incubated at 40 °C with shaking at 150 rpm. After 24 h, the samples were serially diluted (from 10^−1^ to 10^−6^ dilutions) and 100 µl of the diluted samples were spread on antibiotics containing YEPD agar plates (yeast extract: 10.0 g/l, peptone: 20.0 g/l, glucose: 20 g/l and agar: 20.0 g/l), incubated at 40 °C for 48 h. Various yeast-like colonies were picked and screened for thermotolerance and chrome agar screening. The selected yeast colonies were further purified by continuous streaking on YEPD agar plate. Additionally, several known C5 and C6 assimilating yeast strains were procured from the National culture collection of industrial microorganisms (NCIM), Pune, India (Table 1) and included in this study. In addition, two industrial yeasts *Saccharomyces cerevisiae* CEN.PK-122) [30–32] and a commercially available yeast, Angel yeast (Angel Active Dry Ethanol Yeast, Angel Yeast Co. Ltd., Hubei, China) [33–36] were also included in this study. All yeast isolates were maintained on YEPD agar plates and stored at 4 °C.

### Molecular characterization

Selected yeast isolates were characterized by Internal transcribed spacer (ITS) sequencing followed by blast with NCBI database. Genomic DNA was isolated adopting a previously reported method [66] with slight modification. In brief, yeast cells were grown overnight in 5.0 ml YEPD broth and separated by centrifugation at 4700 rcf for 5 min, washed twice with 10.0 ml sterile water followed by 1.0 ml Phosphate buffer saline (PBS). Washed cells were suspended in 500 µl of lysis buffer {(Tris HCl (50 mM, pH 8.0), EDTA (10 mM), NaCl (150 mM), Triton X-100 (1.0% v/v), SDS (1.0% w/v)}, transferred to 2.0-ml Eppendorf tube and incubated at 65 °C for 30 min. 0.5 g glass beads and 500 µl of Phenol: Chloroform: isoamyl alcohol (25:24:1) were added and mixed with cells by vortexing thrice for 30 s, centrifuged at 12,220 rcf for 12 min and the upper layer was transferred into a new Eppendorf tube. Again, 500 µl of PCI was added, mixed thoroughly, centrifuged and upper layer was collected into a new tube containing 1.0 ml of absolute ethanol, and incubated at − 20 °C for 30 min, centrifuged at 11,280 rcf for 20 min at 4 °C, washed the pellet with ice chilled 70% ethanol, dissolved in 100 µl of sterile water and stored at − 20 °C.

ITS region was PCR amplified using ITS1/ITS4 primers and genomic DNA as a template [66] in a thermocycler (Eppendorf, Nexus GSX1, Germany). The reaction was carried out in 50 µl containing 1.0 µl of genomic DNA, 5.0 µl of PCR Taq buffer, 1.0 µl of deoxyribonucleotide triphosphate (dNTP) mix, 2.5 µl of forward primer (ITS1: 5′-TCCGTAGGTGAACCTGCGG-3′), 2.5 µl of reverse primer (ITS4: 5′-TCCTCCGCTTATTGATATGC-3′), 0.5 µl of Taq DNA polymerase (G-Biosciences, USA) and 37.5 µl of sterile water. The PCR conditions were as following: initial denaturation (95 °C for 5 min), 30 cycles of denaturation (95 °C for 30 s), annealing (52 °C for 30 s) and extension (72 °C for 1.0 min), and a final extension (72 °C for 5.0 min). The PCR products were cleaned up using Gene JET PCR Purification Kit (Thermo scientific, Lithuania) and sequenced (Invitrogen BioServices, India). The ITS sequences were analyzed by nucleotide BLAST against the NCBI database. ITS sequences were aligned by ClustalW (a multiple sequence alignment tool) and analyzed phylogenetically by maximum likelihood method using the Tamura-Nei model and 1000 bootstrap replicates employing molecular evolutionary genetics analysis (MEGA) software version 6.0 [21, 67].

### Growth kinetics

The growth kinetics was performed by a micro-cultivation method in a 96-well plate using Liquid Handling System (Tecan, Austria) in YEPD broth at 30 °C and 40 °C, respectively. Briefly, overnight grown yeast cultures were diluted to OD_600_ = 1.0 and 20 µl of each culture was mixed with 180 µl YEPD broth in 96 well plate and OD_600_ was measured at every 30 min of interval up to 24 h. Specific growth rate (*μ*) and doubling times (*T*_d_) were calculated by measuring the time taken in doubling of logarithms values of the OD_600_ of the exponential phase. Effect of elevated temperature on growth was analyzed by comparing doubling time and specific growth rate at 30 °C and 40 °C, respectively.

### Sugar assimilations and inhibitor tolerance

The sugar assimilation profile was evaluated in SD broth {YNB + 2.0% carbon source, (either hexose: d-glucose, mannose, galactose or pentose: d-xylose, l-arabinose or disaccharides: cellobiose, maltose, lactose)}. Effect of inhibitors on cell growth was measured in SD broth supplemented at graded concentrations of furfural (0.5, 1.0 and 1.5 g/l), 5-HMF (1.0, 2.0 and 3.0 g/l), acetic acid {0.2% v/v (0.034 M), 0.3% v/v (0.051 M) and 0.4% v/v (0.068 M)} and ethanol (6.0, 8.0 and 10.0% v/v), respectively. 200 µl of culture (180 µl YEPD broth and 20 µl individual yeast cultures of OD_600_ = 1.0) was mixed in 96-well plate and incubated with shaking at 40 °C, 150 rpm for 24 h and OD_600_ was analyzed using Liquid Handling System (Tecan, Austria). The above-used broth media were filter sterilized (0.2 µm, Millipore). The effect of tested inhibitors on growth was also checked by spotting assay using 3 µl of culture (OD_600_ 0.1) on SD agar plates and selected concentration of all inhibitors (furfural, 5-HMF, Acetic acid and ethanol) individually. The combinatorial effect of inhibitors on yeast growth was also analyzed in silico through Chemogenetic network. To generate this network, the maximum tolerant phenotypes of each tested inhibitor (including 1.5 g/l of furfural, 3.0 g/l of 5-HMF, 0.3% v/v of acetic acid and 10.0% v/v ethanol) were given as input to Cytoscape 3.6.1 software.

### Ploidy analysis

Ploidy was determined by analyzing the DNA content through flow cytometry by adopting the previously described method with slight modifications [25, 68]. In brief, exponentially grown yeast cells were harvested by centrifugation, washed with sterile water and fixed with 70% v/v ethanol (chilled) for 60 min at room temperature. Cells were washed twice with 1.0 ml Na-citrate buffer (50 mM, pH 4.8) and treated with RNase by re-suspension in 500 μl of Na-citrate buffer containing 0.1 mg/ml RNase for 2 h at 37 °C. Propidium iodide (PI) staining was performed by adding 500 μl of PI solution (20 μg/ml, prepared in the Na-citrate buffer) and staining was performed for 18 h at 4 °C in dark. Ethanol- and RNase-treated unstained cells were used as a control for each isolate. 500 μl of cells was exposed to the FACScan instrument (Becton–Dickinson, USA) and fluorescence intensity was analyzed. Ploidy was determined by comparing fluorescence using analytical flow cytometry (FACS) spectra of each isolate with the spectra of reference haploid (CEN.PK-1137D) and diploid (CEN.PK-122).

### Ethanol fermentation

The fermentation with glucose and xylose as carbon sources was performed in 100-ml serum bottles (clear, with stopper and seal) containing 50 ml of fermentation broth (FB) medium (Yeast extract; 5.0 g/l, (NH4)_2_SO_4_; 3.75 g/l, KH_2_PO_4_; 2.1 g/l, CaCl_2_·2H_2_O; 0.5 g/l and MgSO_4_·7H_2_O; 0.375 g/land pH 5.4) supplemented with 10.0% w/v glucose, 30% w/v glucose and 5.0% w/v xylose, respectively. 5.0% (v/v) seed culture of overnight grown yeast cells in YEPD broth was added into 50-ml fermentation broth and incubated at 30 °C and 40 °C, respectively, with continuous shaking at 150 rpm. After 24 h, fermentate was analyzed for unutilized glucose/xylose, and production of ethanol, xylitol, glycerol and acetic acid using HPLC.

### Fermentation of rice straw hydrolysate via SHF

Rice straw (RS) enzymatic hydrolysates were prepared by dilute acid and dilute alkali pretreatment followed by enzymatic saccharification adopting a previously described method [2] with slight modifications. Briefly, RS biomass was mixed with H_2_SO_4_ (2.0% v/v solution) and NaOH (1.0% w/v solution), respectively, at biomass loading of 10.0% w/v, autoclaved at 121 °C for 45 min. The slurry obtained was filtered using muslin cloth and obtained biomass residues were washed with water until neutral pH was achieved and then dried at 45 °C. For saccharification, the dried pre-treated RS biomass (DPRSB) was mixed with Na-citrate buffer (50 mM, pH4.8) at 5.0% w/v loading using 15 FPU cellulase (Sigma-Aldrich, India) per gram of DPRSB in 250-ml screw-capped flasks. The saccharification was performed at 50 °C for 72 h with continuous shaking at 150 rpm. The resulted saccharified slurry and 4× concentrated slurry were augmented with 0.5% w/v yeast extract and used for direct fermentation without detoxification and filtration, employing 5.0% v/v inoculums (containing 1.0 × 10^7^ cells/ml) of overnight grown yeast cells in YEPD broth. The fermentation was carried out at 30 °C and 40 °C for 24 h with shaking at 150 rpm, and production of ethanol, glycerol and acetic acid along with the residual glucose were analyzed by using HPLC.

### Kinetic study of ethanol production

Kinetic study was performed in batch mode using synthetic fermentation media (Yeast extract; 5.0 g/l, (NH4)_2_SO_4_; 3.75 g/l, KH_2_PO_4_; 2.1 g/l, CaCl_2_·2H_2_O; 0.5 g/l, MgSO_4_·7H_2_O; 0.375 g/l and 5.4) containing glucose (100 g/l), acid-pretreated rice straw enzymatic hydrolysate (APRSEH-1) and alkali-pretreated rice straw enzymatic hydrolysate (APRSEH-2) individually. Additionally, acid- and alkali-pretreated RS hydrolysates were supplemented with 0.5% w/v yeast extract. All fermentation media were inoculated with 5.0% v/v overnight grown isolate NGY10 inoculums (containing 1.0 × 10^7^ cells/ml) followed by incubation at 30 °C and 40 °C, respectively, with shaking at 150 rpm for 48 h. Samples were withdrawn from each fermentation media to every 4 h of interval and centrifuged at 9400 rcf for 10 min. The supernatant was analyzed for kinetic parameters such as ethanol concentration, ethanol yield coefficient (Yp/s), ethanol production rate (*Q*_P_), residual sugar, sugar consumption rate (*Q*_S_), cell biomass concentration and cell growth rate (*Q*_X_) for each sample.

### Ethanol production via SSF

SSF was performed at 40 °C using pretreated RS with 5% w/v and 10% w/v solid loading in Na-citrate buffer (50 mM, pH 5.0) employing 15 FPU/g cellulase and 5.0% v/v inoculums of overnight grown isolate NGY10 for 72 h. SSF with pre-saccharification was performed by incubating Na-citrate buffer drenched RS with cellulase at 50 °C for 6.0 h before adding yeast inoculums. Whereas, no exposure to 50 °C was attempted in SSF without pre-saccharification. Sugar consumption and ethanol production were analyzed using HPLC at different time intervals.

### Analytical methods

To estimate glucose, ethanol, various inhibitors (Furfural, 5-HMF and acetic acid), and other metabolites (glycerol, xylitol and acetic acid), 1.0 ml of sample was centrifuged at 9400 rcf for 10 min, supernatants were syringe filtered (by 0.22 µm, Millipore) and analyzed using HPLC (Agilent, 1260 Infinity). For HPLC analysis, refractive index (RI) detector and Aminex HPX 87H (300 × 7.8 mm) column (Bio-Rad, India) were used with mobile phase H_2_SO_4_ (4 mM) at a flow rate of 0.3 ml/min and column temperature 40 °C. The sugar, ethanol and other metabolites were quantified by dividing the peak area of the sample with the peak area of standard (1.0 g/l) at specific retention time.

## Additional files

**Additional file 1.** Comparison of Acetic acid and ethanol tolerance profile of isolate NGY10 with industrial strains (CEN.PK-122 and Angel yeast) at 30 °C.

**Additional file 2.** FACS spectra of yeast isolates for ploidy determination using propidium iodide (PI) staining.

**Additional file 3.** Fermentation profile at 30 °C, 40 °C and 42 °C in SD media containing 100 g/l glucose in 24 h.

**Additional file 4.** Fermentation profile at 30 °C, 40 °C and 42 °C in SD media containing 50.0 g/l pure xylose in 24 h.

## Abbreviations

SHF: separate hydrolysis and fermentation
SSF: simultaneous saccharification and fermentation
SScF: simultaneous saccharification and co-fermentation
CBP: consolidated bio-processing
C6: hexose sugar
C5: pentose sugar
5-HMF: 5-(hydroxymethyl)furfural
LH: lignocellulosic hydrolysate
YEPD: yeast extract peptone dextrose
°C: degree celsius
g/l: gram per litre
rpm: revolution per minutes
µl: micro litre
NCIM: National culture collection of industrial microorganisms
ITS: internal transcribed spacer
DNA: deoxyribonucleic acid
ml: millilitre
HCl: hydrochloric acid
mM: millimolar
EDTA: ethylenediaminetetraacetic acid
NaCl: sodium chloride
SDS: sodium dodecyl sulphate
PCR: polymerase chain reaction
dNTP: deoxyribonucleotide triphosphate
MEGA: molecular evolutionary genetics analysis
nm: nanometer
OD_600_: optical density at 600 nm
SD: synthetic defined
PI: propidium iodide
DPRSB: dried pre-treated RS biomass
FPU: filter paper unit
RS: rice straw
µ: specific growth rate
Yp/s: ethanol yield coefficient
*Q*_P_: ethanol productivity
*Q*_S_: sugar consumption rate
*Q*_X_: cell biomass concentration and cell growth rate
HPLC: high-performance liquid chromatography
RI: refractive index
NCBI: National Center for Biotechnology Information
NCIM: National Collection of Industrial Microorganisms
APRSEH-1: acid-pretreated rice straw enzymatic hydrolysate
APRSEH-2: alkali-pretreated rice straw enzymatic hydrolysate
FACS: fluorescence-activated cell sorting

## References

[1] Hu ML, Zha J, He LW, Lv YJ, Shen MH, Zhong C (2016). Enhanced bioconversion of cellobiose by industrial *Saccharomyces cerevisiae* used for cellulose utilization. Front Microbiol.

[2] Pandey AK, Negi S (2015). Impact of surfactant assisted acid and alkali pretreatment on lignocellulosic structure of pine foliage and optimization of its saccharification parameters using response surface methodology. Bioresour Technol.

[3] Kumar R, Tabatabaei M, Karimi K, Horvath IS (2016). Recent updates on lignocellulosic biomass derived ethanol—a review. Biofuel Res J.

[4] Peris D, Moriarty RV, Alexander WG, Baker EC, Sylvester K, Sardi M (2017). Hybridization and adaptive evolution of diverse *Saccharomyces* species for cellulosic biofuel production. Biotechnol Biofuels.

[5] Binod P, Sindhu R, Singhania RR, Vikram S, Devi L, Nagalakshmi S (2010). Bioethanol production from rice straw: an overview. Bioresour Technol.

[6] Choudhary J, Singh S, Nain L (2017). Bioprospecting thermotolerant ethanologenic yeasts for simultaneous saccharification and fermentation from diverse environments. J Biosci Bioeng.

[7] Rezania S, Fadhil M, Din M, Mohamad SE, Sohaili J, Taib SM (2017). Review on pretreatment methods and ethanol production from cellulosic water hyacinth. BioResources.

[8] Pandey AK, Edgard G, Negi S (2016). Optimization of concomitant production of cellulase and xylanase from *Rhizopus oryzae* SN5 through EVOP-factorial design technique and application in Sorghum Stover based bioethanol production. Renew Energy.

[9] Kang Q, Appels L, Tan T, Dewil R, Kang Q, Appels L (2014). Bioethanol from Lignocellulosic Biomass: Current Findings Determine Research Priorities. Sci World J.

[10] Wingren A, Galbe M, Zacchi G (2008). Techno-economic evaluation of producing ethanol from softwood: comparison of SSF and SHF and identification of bottlenecks. Biotechnol Prog.

[11] Inokuma K, Iwamoto R, Bamba T, Hasunuma T, Kondo A (2017). Improvement of xylose fermentation ability under heat and acid co-stress in *Saccharomyces cerevisiae* using genome shuffling technique. Front Bioeng Biotechnol.

[12] Alfani F, Gallifuoco A, Saporosi A, Spera A, Cantarella M (2000). Comparison of SHF and SSF processes for the bioconversion of steam-exploded wheat straw. J Ind Microbiol Biotechnol.

[13] Olofsson K, Rudolf A, Liden G (2008). Designing simultaneous saccharification and fermentation for improved xylose conversion by a recombinant strain of *Saccharomyces cerevisiae*. J Biotechnol.

[14] Olofsson K, Bertilsson M, Liden G (2008). A short review on SSF—an interesting process option for ethanol production from lignocellulosic feedstocks. Biotechnol Biofuels.

[15] Hasunuma T, Kondo A (2012). Development of yeast cell factories for consolidated bioprocessing of lignocellulose to bioethanol through cell surface engineering. Biotechnol Adv.

[16] Lau MW, Gunawan C, Balan V, Dale BE (2010). Comparing the fermentation performance of *Escherichia coli* KO11, *Saccharomyces cerevisiae* 424A (LNH-ST) and *Zymomonas mobilis* AX101 for cellulosic ethanol production background methods microbial strains. Biotechnol Biofuels.

[17] Caspeta L, Castillo T, Nielsen J (2015). Modifying yeast tolerance to inhibitory conditions of ethanol production processes. Front Bioeng Biotechnol.

[18] Almeida JR, Modig T, Petersson A, Hahn-Hagerdal B, Liden G, Gorwa-Grauslund MF (2007). Increased tolerance and conversion of inhibitors in lignocellulosic hydrolysates by *Saccharomyces cerevisiae*. J Chem Technol Biotechnol.

[19] Jonsson LJ, Alriksson B, Nilvebrant N-O, de Sousa F, Gorton L, Jonsson L (2013). Bioconversion of lignocellulose: inhibitors and detoxification. Biotechnol Biofuels.

[20] Nasir A, Rahman SS, Hossain MM, Choudhury N (2017). Isolation of *Saccharomyces cerevisiae* from pineapple and orange and study of metal’s effectiveness on ethanol production. Eur J Microbiol Immunol.

[21] Chamnipa N, Thanonkeo S, Klanrit P, Thanonkeo P (2018). The potential of the newly isolated thermotolerant yeast *Pichia kudriavzevii* RZ8-1 for high-temperature ethanol production. Brazilian J Microbiol.

[22] Jin YS, Alper H, Yang YT, Stephanopoulos G (2005). Improvement of xylose uptake and ethanol production in recombinant *Saccharomyces cerevisiae* through an inverse metabolic engineering approach. Appl Environ Microbiol.

[23] Chandel AK, da Silva SS, Singh OV (2013). Detoxification of lignocellulose hydrolysates: biochemical and metabolic engineering toward white biotechnology. BioEnergy Res.

[24] Sani RK, Srivastava N, Li S, Sindhu R, Madhavan A, Alphonsa Jose A (2017). Synthetic biology and metabolic engineering approaches and its impact on non-conventional yeast and biofuel production. Front Energy Res.

[25] Dubey R, Jakeer S, Gaur NA (2016). Screening of natural yeast isolates under the effects of stresses associated with second-generation biofuel production. J Biosci Bioeng.

[26] Mishra A, Sharma AK, Sharma S, Mathur AS, Gupta RP, Tuli DK (2016). Lignocellulosic bioethanol production employing newly isolated inhibitor and thermotolerant *Saccharomyces cerevisiae* DBTIOC S24 strain in SSF and SHF. RSC Adv..

[27] Favaro L, Basaglia M, Trento A, Van Rensburg E, Garcia-Aparicio M, Van Zyl WH (2013). Exploring grape marc as trove for new thermotolerant and inhibitor-tolerant *Saccharomyces cerevisiae* strains for second-generation bioethanol production. Biotechnol Biofuels.

[28] Pereira FB, Romani A, Ruiz HA, Teixeira JA, Domingues L (2014). Industrial robust yeast isolates with great potential for fermentation of lignocellulosic biomass. Bioresour Technol.

[29] Pravin Charles MV, Kali A, Joseph NM (2015). Performance of chromogenic media for Candida in rapid presumptive identification of *Candida* species from clinical materials. Pharmacognosy Res.

[30] Schehl B, Müller C, Senn T, Heinisch JJ (2004). A laboratory yeast strain suitable for spirit production. Yeast.

[31] Marques WL, Raghavendran V, Stambuk BU, Gombert AK (2016). Sucrose and *Saccharomyces cerevisiae*: a relationship most sweet. FEMS Yeast Res.

[32] Pereira FB, Guimaraes PMR, Teixeira JA, Domingues L (2011). Robust industrial *Saccharomyces cerevisiae* strains for very high gravity bio-ethanol fermentations. J Biosci Bioeng.

[33] Li BZ, Yuan YJ (2010). Transcriptome shifts in response to furfural and acetic acid in *Saccharomyces cerevisiae*. Appl Microbiol Biotechnol.

[34] Liu ZH, Qin L, Zhu JQ, Li BZ, Yuan YJ (2014). Simultaneous saccharification and fermentation of steam-exploded corn stover at high glucan loading and high temperature. Biotechnol Biofuels.

[35] You Y, Li P, Lei F, Xing Y, Jiang J (2017). Enhancement of ethanol production from green liquor-ethanol-pretreated sugarcane bagasse by glucose-xylose cofermentation at high solid loadings with mixed *Saccharomyces cerevisiae* strains. Biotechnol Biofuels.

[36] Xu K, Gao L, Hassan JU, Zhao Z, Li C, Huo YX (2018). Improving the thermo-tolerance of yeast base on the antioxidant defense system. Chem Eng Sci.

[37] Diezmann S, Cox CJ, Schonian G, Vilgalys RJ, Mitchell TG (2004). Phylogeny and evolution of medical species of *Candida* and related taxa: a multigenic analysis. J Clin Microbiol.

[38] McMillan JD, Boynton BL (1994). Arabinose utilization by xylose-fermenting yeasts and fungi. Appl Biochem Biotechnol.

[39] Hespell RB (1998). Extraction and characterization of hemicellulose from the corn fiber produced by corn wet-milling processes. J Agric Food Chem.

[40] Anderson MJ, Barker SL, Boone C, Measday V (2012). Identification of RCN1 and RSA3 as ethanol-tolerant genes in *Saccharomyces cerevisiae* using a high copy barcoded library. FEMS Yeast Res.

[41] Puig S, Querol A, Barrio E, Perez-Ortin JE (2000). Mitotic recombination and genetic changes in *Saccharomyces cerevisiae* during wine fermentation. Appl Env Microbiol..

[42] Arora R, Behera S, Sharma NK, Kumar S (2015). A new search for thermotolerant yeasts, its characterization and optimization using response surface methodology for ethanol production. Front Microbiol.

[43] Qiu Z, Jiang R (2017). Improving Saccharomyces cerevisiae ethanol production and tolerance via RNA polymerase II subunit Rpb7. Biotechnol Biofuels.

[44] Pais TM, Foulquie-Moreno MR, Hubmann G, Duitama J, Swinnen S, Goovaerts A (2013). Comparative polygenic analysis of maximal ethanol accumulation capacity and tolerance to high ethanol levels of cell proliferation in yeast. PLoS Genet.

[45] Singh A, Bishnoi NR (2012). Enzymatic hydrolysis optimization of microwave alkali pretreated wheat straw and ethanol production by yeast. Bioresour Technol.

[46] Jung YH, Park HM, Kim IJ, Park YC, Seo JH, Kim KH (2014). One-pot pretreatment, saccharification and ethanol fermentation of lignocellulose based on acid-base mixture pretreatment. RSC Adv.

[47] Wang YZ, Liao Q, Lv FL, Zhu X, Ran Y, Hou CJ (2015). Solid simultaneous saccharification and fermentation of rice straw for bioethanol production using nitrogen gas stripping. RSC Adv.

[48] Saini JK, Agrawal R, Satlewal A, Saini R, Gupta R, Mathur A (2015). Second generation bioethanol production at high gravity of pilot-scale pretreated wheat straw employing newly isolated thermotolerant yeast *Kluyveromyces marxianus* DBTIOC-35. RSC Adv..

[49] Gasch AP, Spellman PT, Kao CM, Carmel-Harel O, Eisen MB, Storz G (2000). Genomic expression programs in the response of yeast cells to environmental changes. Mol Biol Cell.

[50] Lu C, Brauer MJ, Botstein D (2008). Slow growth induces heat-shock resistance in normal and respiratory-deficient yeast. Mol Biol Cell.

[51] Iwaki A, Kawai T, Yamamoto Y, Izawa S (2013). Biomass conversion inhibitors furfural and 5-hydroxymethylfurfural induce formation of messenger RNP granules and attenuate translation activity in *Saccharomyces cerevisiae*. Appl Environ Microbiol.

[52] Zhao L, Liu DXW (2007). Effect of several factors on peracetic acid pretreatment of sugarcane bagasse for enzymatic hydrolysis. J Chem Technol Biotechnol.

[53] Zha Y, Westerhuis JA, Muilwijk B, Overkamp KM, Nijmeijer BM, Coulier L (2014). Identifying inhibitory compounds in lignocellulosic biomass hydrolysates using an exometabolomics approach. BMC Biotechnol.

[54] Chen Y, Stabryla L, Wei N (2016). Improved acetic acid resistance in *Saccharomyces cerevisiae* by overexpression of the WHI2 gene identified through inverse metabolic engineering. Appl Environ Microbiol.

[55] Wallace-Salinas V, Gorwa-Grauslund MF (2013). Adaptive evolution of an industrial strain of *Saccharomyces cerevisiae* for combined tolerance to inhibitors and temperature. Biotechnol Biofuels.

[56] Aarnio TH, Suihko ML, Kauppinen VS (1991). Isolation of acetic acid-tolerant Baker’s yeast variants in a turbidostat. Appl Biochem Biotechnol.

[57] Teixeira MC, Godinho CP, Cabrito TR, Mira NP, Sa-Correia I (2012). Increased expression of the yeast multidrug resistance ABC transporter Pdr18 leads to increased ethanol tolerance and ethanol production in high gravity alcoholic fermentation. Microb Cell Fact.

[58] Alper H, Moxley J, Nevoigt E, Fink GR, Stephanopoulos G (2006). Engineering yeast transcription machinery for improved ethanol tolerance and production. Science.

[59] Thammasittirong SN-R, Thirasaktana T, Thammasittirong A, Srisodsuk M (2013). Improvement of ethanol production by ethanol-tolerant *Saccharomyces cerevisiae* UVNR56. Springerplus.

[60] Palma M, Guerreiro JF, Sa-Correia I (2018). Adaptive Response and Tolerance to Acetic Acid in *Saccharomyces cerevisiae* and *Zygosaccharomyces bailii*: a Physiological Genomics Perspective. Front Microbiol.

[61] Stanley D, Bandara A, Fraser S, Chambers PJ, Stanley GA (2010). The ethanol stress response and ethanol tolerance of *Saccharomyces cerevisiae*. J Appl Microbiol.

[62] Wu X, Zhang L, Jin X, Fang Y, Zhang K, Qi L (2016). Deletion of JJJ1 improves acetic acid tolerance and bioethanol fermentation performance of *Saccharomyces cerevisiae* strains. Biotechnol Lett.

[63] Kim HS, Kim NR, Choi W (2011). Total fatty acid content of the plasma membrane of *Saccharomyces cerevisiae* is more responsible for ethanol tolerance than the degree of unsaturation. Biotechnol Lett.

[64] You KM, Rosenfield C, Knipple DC (2003). Ethanol tolerance in the yeast *Saccharomyces cerevisiae* is dependent on cellular oleic acid content. Appl Environ Microbiol.

[65] Wang J, Chae M, Sauvageau D, Bressler DC (2017). Improving ethanol productivity through self-cycling fermentation of yeast: a proof of concept. Biotechnol Biofuels.

[66] Vasdinyei R, Deak T (2003). Characterization of yeast isolates originating from Hungarian dairy products using traditional and molecular identification techniques. Int J Food Microbiol.

[67] Pandey AK, Sarada DVL, Kumar A (2016). Microbial decolorization and degradation of reactive red 198 azo dye by a newly isolated *Alkaligenes* species. Proc Natl Acad Sci India Sect B Biol Sci.

[68] Demeke MM, Dietz H, Li Y, Foulquie-Moreno MR, Mutturi S, Deprez S (2013). Development of a D-xylose fermenting and inhibitor tolerant industrial *Saccharomyces cerevisiae* strain with high performance in lignocellulose hydrolysates using metabolic and evolutionary engineering. Biotechnol Biofuels.

