# Supplementary material for: Evaluation of divergent yeast genera for fermentation-associated stresses and identification of a robust sugarcane distillery waste isolate Saccharomyces cerevisiae NGY10 for lignocellulosic ethanol production in SHF and SSF
Source: Biotechnol Biofuels. 2019 Feb 27;12:40. doi: 10.1186/s13068-019-1379-x (PMC6391804; doi:10.1186/s13068-019-1379-x)
Supplement: Supplementary file 1 — Additional file 1. Comparison of Acetic acid and ethanol tolerance profile of isolate NGY10 with industrial strains (CEN.PK-122 and Angel yeast) at 30 °C. [file 13068_2019_1379_MOESM1_ESM.pptx]

## Slide 1
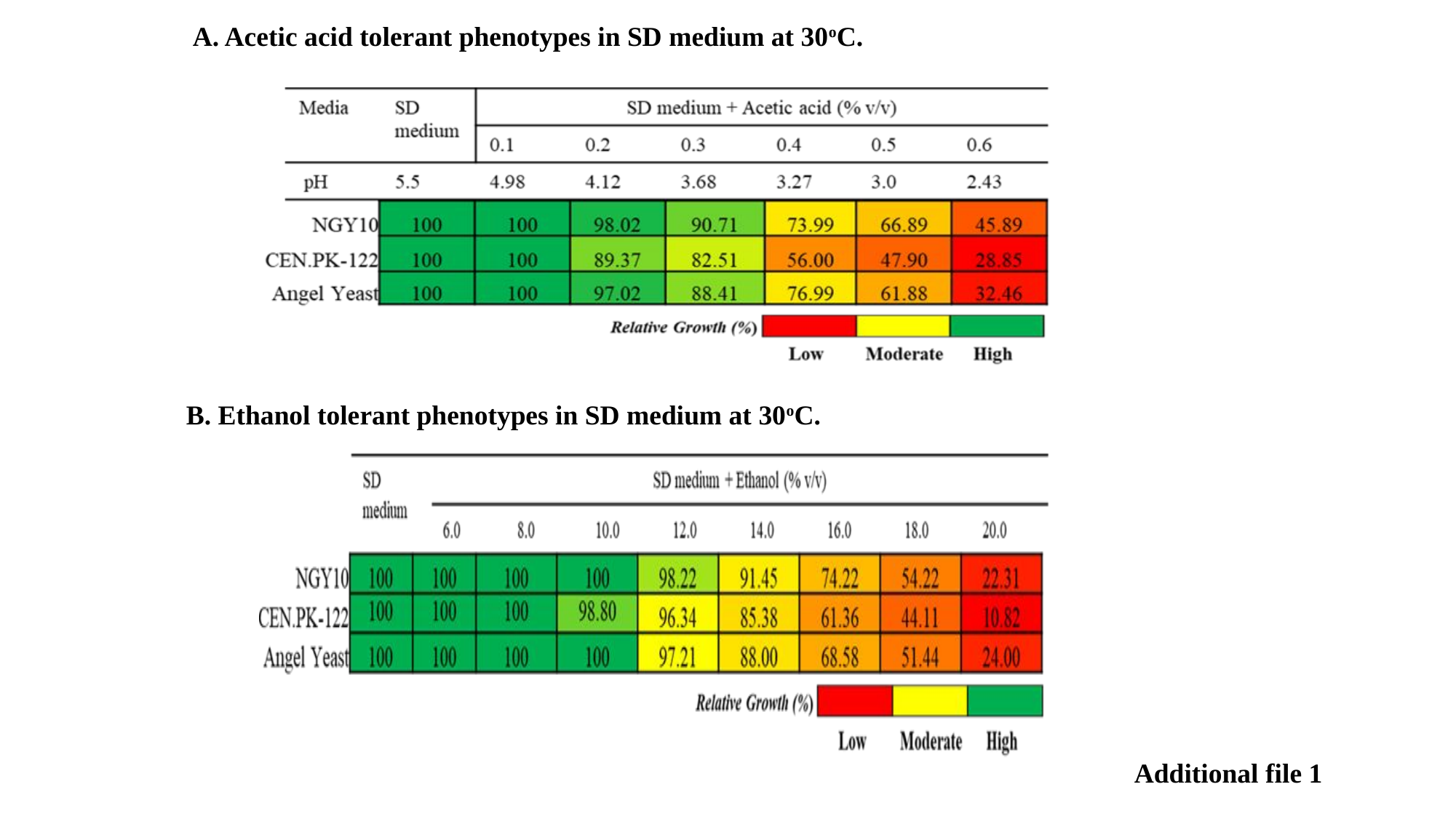

A. Acetic acid tolerant phenotypes in SD medium at 30oC.
B. Ethanol tolerant phenotypes in SD medium at 30oC.
Additional file 1
