# Supplementary figures and images for: Evaluation of divergent yeast genera for fermentation-associated stresses and identification of a robust sugarcane distillery waste isolate Saccharomyces cerevisiae NGY10 for lignocellulosic ethanol production in SHF and SSF
Source: Biotechnol Biofuels. 2019 Feb 27;12:40. doi: 10.1186/s13068-019-1379-x (PMC6391804; doi:10.1186/s13068-019-1379-x)

## Slide 1
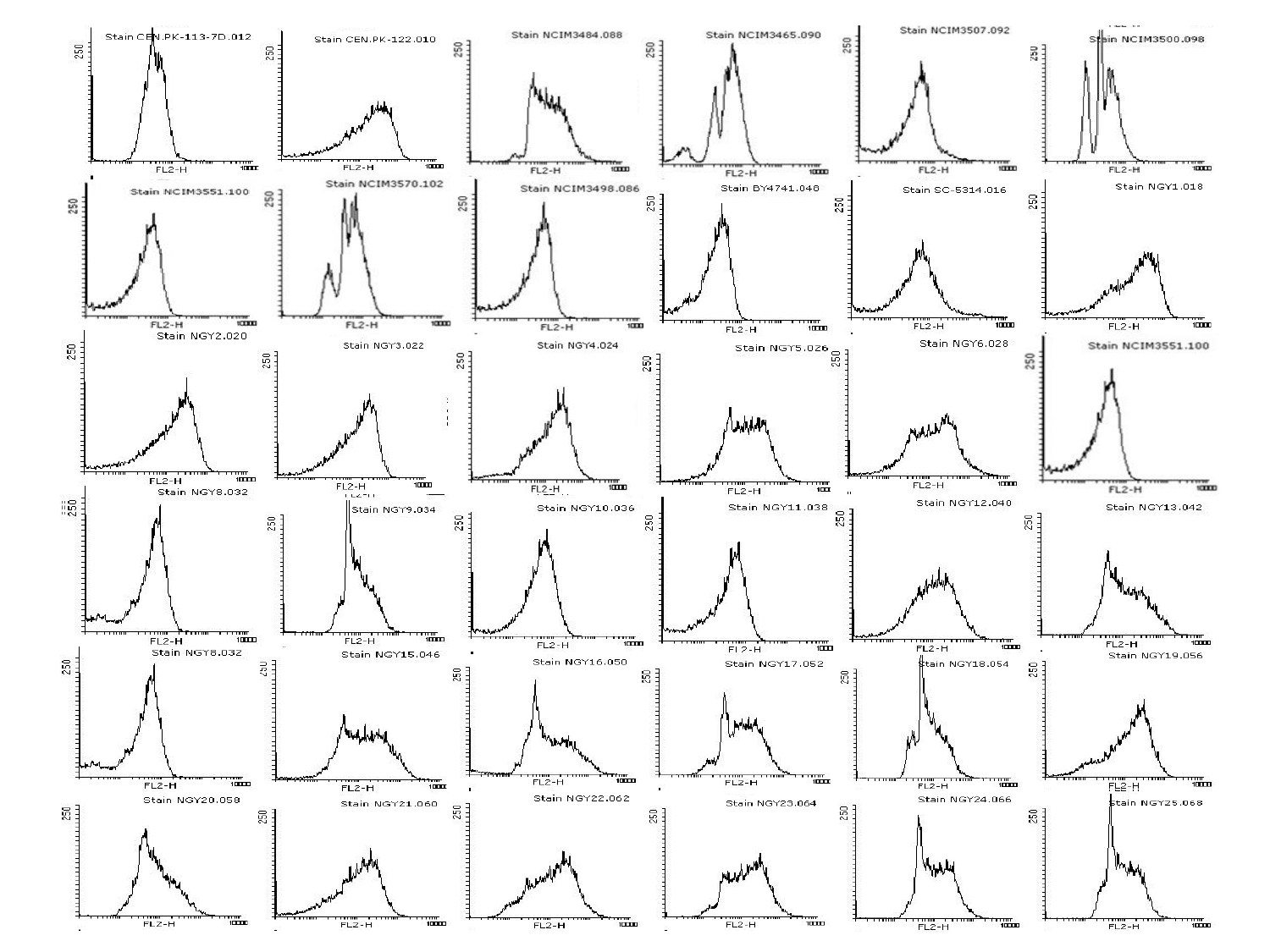

Supplement: Supplementary file 2 — Additional file 2. FACS spectra of yeast isolates for ploidy determination using propidium iodide (PI) staining. [file 13068_2019_1379_MOESM2_ESM.pptx]
