# Supplementary material for: Evaluation of divergent yeast genera for fermentation-associated stresses and identification of a robust sugarcane distillery waste isolate Saccharomyces cerevisiae NGY10 for lignocellulosic ethanol production in SHF and SSF
Source: Biotechnol Biofuels. 2019 Feb 27;12:40. doi: 10.1186/s13068-019-1379-x (PMC6391804; doi:10.1186/s13068-019-1379-x)
Supplement: Supplementary file 3 — Additional file 3. Fermentation profile at 30 °C, 40 °C and 42 °C in SD media containing 100 g/l glucose in 24 h. [file 13068_2019_1379_MOESM3_ESM.docx]

**Additional file 3: Fermentation performances of selected yeast strains at 30^o^C, 40^o^C and 42^o^C in SD media containing 100 g/l glucose in 24 hours.**

| **S. No.** | **Yeast Strains** | **Ploidy** | **30^o^C** | | | | **40^o^C** | | | | **42^o^C** | | | |
| --- | --- | --- | --- | --- | --- | --- | --- | --- | --- | --- | --- | --- | --- | --- |
|  |  |  | **Ethanol concentration**  **(g/l)** | **Ethanol Yield**  **(g/g)** | **Ethanol Productivity (g/l//h)** | **Ethanol efficiency (%)** | **Ethanol concentration**  **(g/l)** | **Ethanol Yield**  **(g/g)** | **Ethanol Productivity (g/l//h)** | **Ethanol efficiency (%)** | **Ethanol concentration**  **(g/l)** | **Ethanol Yield**  **(g/g)** | **Ethanol Productivity (g/l//h)** | **Ethanol efficiency (%)** |
|  | Angel yeast | - | 48.94 ± 1.38^a^ | 0.489 | 2.04 | 95.69 | 45.91 ± 1.45 | 0.471 | 1.91 2 | 92.17 | - | - | - | - |
|  | *S. cerevisiae* CEN-PK-122 | Diploid | 48.79 ± 0.61* | 0.488 | 2.032 | 95.5 | 41.58 ± 1.08 | 0.43 | 1.732 | 84.148 | 36.12 | 0.37 | 1.50 | 72.49 |
|  | *S. cerevisiae* NCIM 3570 | Aneuploid | 35.30 | 0.49 | 0.74 | 97.71 | 14.1 | 0.19 | 0.292 | 38.92 | 13.47 | 0.19 | 0.56 | 37.30 |
|  | *S. cerevisiae* NGY1 | Aneuploid | 47.59 ± 2.34 | 0.485 | 1.983 | 94.91 | 47.59 ± 2.34 | 0.485 | 1.983 | 94.91 | 35.69 | 0.35 | 1.48 | 69.88 |
|  | *S. cerevisiae* NGY10 | Diploid | 49.77 ± 0.34 | 0.497 | 2.073 | 97.397 | 46.81 ± 3.11 | 0.478 | 1.950 | 93.542 | 40.11 | 0.40 | 1.67 | 79.40 |
|  | *K. marxianus* NCIM 3465 | Haploid | 42.19 ± 3.08 | 0.48 | 1.757 | 93.933 | 41.22 ± 2.87 | 0.45 | 1.7175 | 88.062 | 38.42 | 0.39 | 1.60 | 76.88 |
|  | *K. marxianus* NGY8 | Haploid | 46.50 ± 0.99 | 0.473 | 1.937 | 92.563 | 42.46± 2.00 | 0.461 | 1.769 | 90.215 | 43.5 | 0.43 | 1.81 | 85.83 |
|  | *K. lactis* NCIM 3551 | Haploid | 34.91 | 0.46 | 0.73 | 90.99 | 14.89 | 0.2 | 0.31 | 38.81 | 18.78 | 0.25 | 0.78 | 48.95 |
|  | *S. stipitis* NCIM 3507 | Aneuploid | 14.15 | 0.16 | 0.294 | 32.3 | 2.68 | 0.03 | 0.05 | 6.11 | 1.41 | 0.01 | 0.05 | 3.21 |
|  | *S. stipitis* NCIM 3498 | Haploid | 4.15 | 0.04 | 0.086 | 8.5 | 0.85 | 0.008 | 0.018 | 1.752 | 1.1 | 0.01 | 0.04 | 2.24 |
|  | *C. shehatae* NCIM 3500 | Aneuploid | 26.42 | 0.35 | 0.55 | 70.3 | 31.44 | 0.42 | 0.654 | 83.62 | 22.36 | 0.30 | 0.93 | 59.47 |
|  | *C. lusitaniae* NCIM 3484 | Diploid | 39.31 | 0.48 | 0.818 | 95.34 | 10.44 | 0.13 | 0.217 | 25.31 | 10.88 | 0.13 | 0.45 | 26.32 |
|  | *C. albicans* SC5314 | Diploid | 44.63 | 0.45 | 0.92 | 89.02 | 34.73 | 0.35 | 0.72 | 69.27 | 28.56 | 0.29 | 1.19 | 56.97 |
|  | *W. anomalus* NGY2 | Diploid | 22.11 | 0.31 | 0.46 | 62.1 | 11.82 | 0.16 | 0.25 | 33.2 | 4.792 | 0.06 | 0.19 | 13.45 |
|  | *O. thermophila* NGY11 | Aneuploid | 7.94 | 0.27 | 0.16 | 54.28 | 13.92 | 0.48 | 0.28 | 95.08 | 11.41 | 0.39 | 0.47 | 77.99 |
|  | *C. glabrata* CBS138 | Haploid | 43.63 ± 2.23 | 0.472 | 1.817 | 92.367 | 42.72 ± 2.55 | 0.459 | 1.78 | 89.823 | 37.89 | 0.38 | 1.57 | 75.61 |
|  | *C. glabrata* NGY7 | Haploid | 47.81 ± 2.11 | 0.48 | 1.992 | 93.933 | 46.09 ± 1.44 | 0.46 | 1.92 | 90.02 | 44.02 | 0.44 | 1.83 | 87.14 |
|  | *C. glabrata* NGY14 | Haploid | 46.06 | 0.46 | 0.95 | 90.29 | 44.61 | 0.44 | 0.93 | 87.46 | 41.09 | 0.41 | 1.71 | 80.55 |
|  | *P. kudriavzevii* NGY12 | Diploid | 45.63 ± 2.71 | 0.469 | 1.901 | 91.78 | 43.60 ± 1.88 | 0.455 | 1.816 | 89.04 | 36.32 | 0.37 | 1.51 | 73.51 |
|  | *P. kudriavzevii* NGY13 | Diploid | 43.89 | 0.49 | 0.91 | 96.61 | 32.56 | 0.36 | 0.678 | 71.66 | 30.97 | 0.34 | 1.29 | 68.18 |
|  | *P. kudriavzevii* NGY15 | Diploid | 42.23 | 0.48 | 0.87 | 95.50 | 40.01 | 0.46 | 0.83 | 90.47 | 38.38 | 0.44 | 1.59 | 86.81 |
|  | *P. kudriavzevii* NGY16 | Diploid | 37.35 | 0.46 | 0.77 | 91.54 | 31.21 | 0.39 | 0.65 | 76.5 | 30.01 | 0.37 | 1.25 | 73.56 |
|  | *P. kudriavzevii* NGY20 | Diploid | 43.44 ± 1.38 | 0.47 | 1.81 | 91.976 | 42.12 ± 1.45 | 0.462 | 1.755 | 90.41 | 40.63 | 0.44 | 1.69 | 86.26 |
|  | *C. dubliniensis* NGY5 | Diploid | 40.13 | 0.44 | 0.84 | 86.41 | 36.17 | 0.39 | 0.75 | 77.89 | 18.74 | 0.20 | 0.78 | 40.36 |
|  | *C. tropicalis* NGY3 | Diploid | 48.17 | 0.48 | 1.00 | 94.92 | 32.85 | 0.33 | 0.7 | 64.72 | 25.09 | 0.25 | 1.04 | 49.45 |
|  | *C. tropicalis* NGY4 | Diploid | 48.79 | 0.49 | 1.02 | 96.01 | 41.58 | 0.41 | 0.86 | 81.83 | 32.19 | 0.32 | 1.34 | 63.33 |
|  | *C. tropicalis* NGY6 | Diploid | 43.46 | 0.45 | 0.91 | 87.97 | 33.84 | 0.34 | 0.7 | 68.48 | 20.89 | 0.21 | 0.87 | 42.28 |
|  | *C. tropicalis* NGY9 | Diploid | 44.11 | 0.45 | 0.91 | 89.12 | 33.43 | 0.34 | 0.69 | 67.54 | 20.15 | 0.20 | 0.83 | 40.72 |
|  | *C. tropicalis* NGY17 | Diploid | 43.90 | 0.50 | 1.03 | 98.05 | 39.29 | 0.39 | 0.82 | 77.2 | 24.69 | 0.24 | 1.02 | 48.51 |
|  | *C. tropicalis* NGY18 | Diploid | 45.67 | 0.48 | 0.95 | 94.60 | 32.98 | 0.34 | 0.69 | 68.33 | 29.26 | 0.30 | 1.21 | 60.61 |
|  | *C. tropicalis* NGY19 | Diploid | 41.49 ± 1.96 | 0.43 | 1.81 | 84.15 | 41.77 ± 1.98 | 0.429 | 1.740 | 84.0 | 28.45 | 0.29 | 1.18 | 58.18 |
|  | *C. tropicalis* NGY21 | Diploid | 41.59 | 0.46 | 0.86 | 90.24 | 41.50 | 0.46 | 0.86 | 90.04 | 26.88 | 0.29 | 1.12 | 58.33 |
|  | *C. tropicalis* NGY22 | Diploid | 47.92 | 0.49 | 0.99 | 96.76 | 38.15 | 0.39 | 0.79 | 77.05 | 21.27 | 0.21 | 0.88 | 42.95 |
|  | *C. tropicalis* NGY23 | Diploid | 39.09 | 0.42 | 0.813 | 82.32 | 34.86 | 0.37 | 0.73 | 73.42 | 19.37 | 0.20 | 0.80 | 40.79 |
|  | *C. tropicalis* NGY24 | Diploid | 38.92 | 0.41 | 0.813 | 80.22 | 35.70 | 0.37 | 0.74 | 73.59 | 23.65 | 0.24 | 0.98 | 48.75 |
|  | *C. tropicalis* NGY25 | Diploid | 38.02 | 0.47 | 0.79 | 91.51 | 32.68 | 0.4 | 0.68 | 78.64 | 22.41 | 0.27 | 0.93 | 53.94 |

* Mean ± standard deviation, n=3; Fermentation volume: 50 ml; pH-5.4; inoculums 5.0 % v/v ≈1.0 x 10^7^ cells/ml
