# Supplementary material for: Evaluation of divergent yeast genera for fermentation-associated stresses and identification of a robust sugarcane distillery waste isolate Saccharomyces cerevisiae NGY10 for lignocellulosic ethanol production in SHF and SSF
Source: Biotechnol Biofuels. 2019 Feb 27;12:40. doi: 10.1186/s13068-019-1379-x (PMC6391804; doi:10.1186/s13068-019-1379-x)
Supplement: Supplementary file 4 — Additional file 4. Fermentation profile at 30 °C, 40 °C and 42 °C in SD media containing 50.0 g/l pure xylose in 24 h. [file 13068_2019_1379_MOESM4_ESM.docx]

| S. No. | **Strains** | **30^o^C** | | **40^o^C** | | **42^o^C** | |
| --- | --- | --- | --- | --- | --- | --- | --- |
|  |  | **Ethanol**  **Concentration (g/l)** | **Xylitol Concentration**  **(g/l)** | **Ethanol**  **Concentration**  **(g/l)** | **Xylitol Concentration**  **(g/l)** | **Ethanol**  **Concentration**  **(g/l)** | **Xylitol Concentration**  **(g/l)** |
|  | Angel yeast | 0 | 0 | 0 | 0 | 0 | 0 |
|  | *S. cerevisiae* CEN-PK-122 | 0 | 0 | 0 | 0 | 0 | 0 |
|  | *S. cerevisiae* NCIM 3570 | 0 | 0 | 0 | 0 | 0 | 0 |
|  | *S. cerevisiae* NGY1 | 0 | 0 |  | 0 |  | 0 |
|  | *S. cerevisiae* NGY10 | 0 | 0 | 0 | 0 | 0 | 0 |
|  | *K. marxianus* NCIM 3465 | 1.262 ± 0.04* | 1.54 ± 0.06 | 0 | 0 | 0 | 0 |
|  | *K. marxianus* NGY8 | 1.525 ± 0.013 | 1.502 ± 0.12 | 1.48 ± 0.025 | 4.39 ± 0.16 | 0 | 4.54 ± 0.31 |
|  | *K. lactis* NCIM 3551 | 0.327 ± 0.01 | 0.22 ± 0.01 | 0 | 0 | 0 | 0 |
|  | *S. stipitis* NCIM 3507 | 6.038 ± 0.09 | 0.018 ± 0.00 | 3.37 ± 0.128 | 0.231± 0.025 | 3.11 ± 0.121 | 0.511± 0.04 |
|  | *S. stipitis* NCIM 3498 | 6.393 ± 0.12 | 0.022 ± 0.00 | 3.75 ± 0.133 | 0 | 3.21 ± 0.13 | 0 |
|  | *C. shehatae* NCIM 3500 | 5.821 ± 0.02 | 1.651 ± 0.024 | 3.74 ± 0.016 | 0 | 0 | 0 |
|  | *C. lusitaniae* NCIM 3484 | 1.509 ± 0.01 | 1.653 ± 0.08 | 0.601± 0.023 | 0.936± 0.04 | 0.302± 0.09 | 1.13± 0.025 |
|  | *C. albicans* SC5314 | 0.476 ± 0.023 | 1.021 ± 0.058 | 0 | 3.11 ± 0.18 | 0 | 3.35 ± 0.15 |
|  | *W. anomalus* NGY2 | 0.704 ± 0.03 | 0.96 ± 0.04 | 0.614 ± 0.019 | 2.12 ± 0.26 | 0.631 ± 0.016 | 3.15 ± 0.09 |
|  | *O. thermophila* NGY11 | 0.445 ± 0.03 | 1.08 ± 0.07 | 0 | 3.44 ± 0.03 | 0 | 4.76 ± 0.09 |
|  | *C. glabrata* CBS138 | 0 | 0 | 0 | 3.39 ± 0.13 | 0 | 3.51 ± 0.19 |
|  | *C. glabrata* NGY7 | 0.093 ± 0.00 | 0.968 ± 0.075 | 0 | 4.44 ± 0.32 | 0 | 4.66 ± 0.35 |
|  | *C. glabrata* NGY14 | 0.11 ± 0.006 | 0.459 ± 0.08 | 0 | 5.04 ± 0.24 | 0 | 5.13 ± 0.22 |
|  | *P. kudriavzevii* NGY12 | 0.544± 0.025 | 0.67± 0.04 | 0.444± 0.025 | 3.3± 0.04 | 0.41± 0.025 | 4.31 ± 0.04 |
|  | *P. kudriavzevii* NGY13 | 0 | 0 | 0 | 0 | 0 | 0 |
|  | *P. kudriavzevii* NGY15 | 0 | 0 | 0 | 0 | 0 | 0 |
|  | *P. kudriavzevii* NGY16 | 0.175 ± 0.01 | 0.079 ± 0.017 | 0 | 0 | 0 | 0 |
|  | *P. kudriavzevii* NGY20 | 0 | 0 | 0 | 0 | 0 | 0 |
|  | *C. dubliniensis* NGY5 | 0.535 ± 0.03 | 0.981± 0.051 | 0 | 4.22 ± 0.22 | 0 | 4.55 ± 0.31 |
|  | *C. tropicalis* NGY3 | 0.752 ± 0.025 | 0.892 ± 0.035 | 0.522 ± 0.122 | 6.14 ± 0.19 | 0.461 ± 0.031 | 6.78 ± 0.22 |
|  | *C. tropicalis* NGY4 | 0.54 ± 0.023 | 1.057 ± 0.055 | 0 | 6.46 ± 0.19 | 0 | 6.79 ± 0.26 |
|  | *C. tropicalis* NGY6 | 0.513 ± 0.024 | 0.980 ± 0.031 | 0 | 4.13 ± 0.20 | 0 | 4.44 ± 0.23 |
|  | *C. tropicalis* NGY9 | 0.574 ± 0.025 | 1.017 ± 0.06 | 0 | 6.88 ± 0.15 | 0 | 7.55 ± 0.31 |
|  | *C. tropicalis* NGY17 | 0.704 ± 0.03 | 1.191 ± 0.09 | 0.966 ± 0.118 | 6.92 ± 0.18 | 0.981 ± 0.019 | 7.33 ± 0.29 |
|  | *C. tropicalis* NGY18 | 0.636 ± 0.027 | 0.927 ± 0.045 | 0.626 ± 0.051 | 5.47 ± 0.32 | 0.584 ± 0.06 | 5.78 ± 0.31 |
|  | *C. tropicalis* NGY19 | 0.646 ± 0.026 | 1.336 ± 0.068 | 0.53 ± 0.029 | 8.14 ± 0.36 | 0.48 ± 0.017 | 8.69 ± 0.19 |
|  | *C. tropicalis* NGY21 | 0.575 ± 0.021 | 1.085 ± 0.052 | 0 | 5.97 ± 0.26 | 0 | 6.39 ± 0.18 |
|  | *C. tropicalis* NGY22 | 0.794 ± 0.03 | 1.683 ± 0.051 | 0.541 ± 0.028 | 7.82 ± 0.31 | 0.521 ± 0.035 | 8.05 ± 0.34 |
|  | *C. tropicalis* NGY23 | 0.42 ± 0.02 | 1.416 ± 0.073 | 0 | 8.33 ± 0. 21 | 0 | 8.66 ± 0.33 |
|  | *C. tropicalis* NGY24 | 0.606 ± 0.031 | 0.957 ± 0.042 | 0.438 ± 0.116 | 5.35 ± 0.15 | 0.41 ± 0.07 | 6.11 ± 0.26 |
|  | *C. tropicalis* NGY25 | 0.765 ± 0.026 | 1.229 ± 0.082 | 0.725 ± 0.08 | 7.69 ± 0.22 | 0.46 ± 0.061 | 8.34 ± 0.28 |

**Additional file 4: Fermentation performances of selected yeast strains at 30^o^C, 40^o^Cand 42^o^C in SD media containing 50.0 g/l pure xylose in 24 hours.**

* Mean ± standard deviation, n=3; Fermentation volume: 50 ml; pH-5.4; inoculums 5.0 % v/v ≈1.0 x 10^7^ cells/ml
